# Report of the Madras Medical Board on Epidemic Cholera. Extracted Verbatim from the Original Document, Drawn up by Order of Government, 1824

**Published:** 1832-04-01

**Authors:** 


### MR. CAMERON TO DR. JAMES JOHNSON.

*Royal Hill, Greenwich, March 17th,* 1832.

Sir,—Although I have not the pleasure of being personally acquainted with you, I have long known you through the medium of your writings. In 1817, when I commenced the practice of my profession in India, as a young Surgeon in the Navy, the principal guide I had to direct me, was your work on the Diseases of Warm Climates; and I am very happy to say that, by attending to it, my treatment of disease was particularly successful. Influenced by motives of partiality, both on this account, and because your ideas of the present epidemic seem to correspond nearly with my own, I have to enclose for your perusal, first a printed copy of a report which I made, some time ago, to the Commissioners for Victualling His

Majesty's Navy, &c., on scorbutic diseases, and secondly, a copy of a letter I had the honour of addressing to the "Central Board of Health" on the prevailing epidemic. I am aware that I will have much difficulty in establishing the doctrine which my letter treats of, and solely because the nature of scorbutic affections, in their earlier stages, is so obscure, and so little known;—but I feel confident that I shall succeed eventually; though, perhaps, too late. This is a matter of great importance, because the treatment of the disease, called the "ASIATIC CHOLERA" now prevailing in this country, is far from successful; and how can it be otherwise, when a debilitated individual, who has been affected with scorbutic diarrhœa for a week or ten days, with not more than two or three pounds of blood in his system, and while in the highest state of collapse from exhaustion, even in articulo mortis, is subjected to the operation of the lancet, and to mustard or salt emetics?!! No medical man ever took the lancet in hand with greater confidence and satisfaction, than I did in the cholera of India; and I shall add, that none ever did so with greater success; but it will not do in the scorbutic dysenteries and diarrhœas, &c., at present prevailing in this country.

I am, Sir, with much esteem,
Your most obedient Servant,
CHARLES CAMERON,
*Surgeon R. N.*

(Copy) *Upper George Street, Greenwich, March* 10*th*, 1832.

SIR,—Permit me to state to you, that in my capacity of Surgeon in His Majesty's Navy, I have had various opportunities of witnessing the disease in the East Indies, which at present occupies so much of the attention of the public as well as of the profession; and I have to request you will please, should such a step not be informal, or contrary to etiquette, to lay this statement before "the Central Board of Health" for their consideration.

With a view to point out the claims which I have to the attention of the Board, I have to represent that I was on service in India during the years 1817 and 1818, when the cholera was so prevalent in that country, and saw a good deal of the disease. I have further witnessed the cholera in India at two different periods since that time, viz., in 1826 and 1830, and after much attention to its history and character, and having also seen that disease called Asiatic or Indian Cholera now prevailing in London, I have come to the following conclusions-namely:

That, the cholera I saw in India, and that which I lately witnessed in London, are not the same disease.

That, the disease now in London, although different in some respects, is of a nature similar to, and arising from the same cause as that which occurred in the Penitentiary in the years 1823-4.

That, although there exists some difference in the descriptions given of the present epidemic and the account of the diseases of the Penitentiary published in 1826 by Dr. Latham, they do not militate against the similarity of the two diseases, but are easily reconciled.

That the disorders of the Penitentiary did, and that the present epidemic does, derive their most formidable symptoms and fatal character, if not their existence, from *a scorbutic taint of the system of the patient,* caused by improper diet or deficient nourishment.

That although the symptoms or signs of the scorbutic diathesis are so obscure as not to be generally noticed, yet post-mortem examinations of the existing cholera present the same appearances that have been observed in scorbutic diseases affecting the alimentary canal.

As it would, perhaps, be improper, if not impossible, for me to point out in the limits of this letter, the similarity or identity of the two diseases by a comparison of all the symptoms, I will confine myself to some remarks on the necroscopic appearances only. I feel the less regret in being limited to this point, as most of the advocates for the identity of Indian cholera, and what is so called in this country, have lately abandoned the outworks and have taken up their position in the strong hold of pathology (see Lancet of the 25th ultimo, page 775,) to prove that the two diseases are one and the same, and that it is new to England. I will, therefore, take a concise view of the pathological data on which so much stress is laid, and will arrange the principal appearances noticed in cholera, and draw a parallel between them and those observed in the dissection of scorbutic cases where the disease chiefly affected the alimentary canal.

| *Pathological data, claimed as being characteristic of Cholera.* | *Pathological data observed in Scurvy, chiefly from the appearances in the diseases of the Penitentiary in* 1823-4. |
| --- | --- |
| The intestinal canal pale, without inflammation or congestion. Black patches in the mucous membrane. | "They" (the patients, says Dr. Latham) "became pale, languid, thin, and feeble."—"Small vascular patches were found in the mucous membrane of the intestines and nothing more."—"The only traces of disease discovered were three or four spots of ecchymosis without any appearance of vascularity or inflammation in their neighbourhood."—"Ecchymosis" and "vascular patches" were frequent. |
| Ulceration of the intestines. | "They" (the morbid appearances of the intestines) "were principally of three kinds, ecchymosis, congestion of the small blood-vessels, and ulceration; the two first belonging exclusively to the mucous membrane, the last beginning in the mucous membrane, but subsequently extending to contiguous parts." |
| A congee-looking fluid passed by stool, and found in the intestinal canal. | The stools occasionally consisted of "some turbid colourless fluid."—"There was every degree and species of flux that was ever seen or described, from the cholera of India to the autumnal visitant of our own country." |
| Blue colour of various parts. | "There were large blotches of an irregular shape, and of such an extent as to leave the legs, arms, thighs, or buttocks, almost uniformly livid, with hardly an appearance of their natural complexion." "Livid or blue," it is worthy of remark, is a term used by Dr. Latham. |
| Congestion and dark colour of the liver, and occasionally congestion of the brain. | "Upon dissection we found the vessels of the brain slightly turgid with blood." (This patient died apoplectic; and of course congestion of the liver will take place when the disease, as it generally does, assumes a congestive character.) |
| Brown or black condition of the blood. | All writers on scurvy have described the blood as of a brown or black colour, and I have no hesitation in saying, from much experience, that this is the origo mali—the very essence of the disease. |

I have thus endeavoured to point out the identity of the pathological appearances observed in the present epidemic, and in the scorbutic disorders of some years back, from Dr. Latham's account of the diseases of the Penitentiary; and I think I have mentioned every fact in this department which those who advocate that cholera is a disease sui generis, and new to this country, will esteem of any importance, with the following exception.

I take it from the description of Moreau de Jonnes, and it seems to be the "pearl of great price." "The most striking peculiarity is," says he, "on the entire, the existence in the alimentary canal of an argillaceous substance, apparently deposited by the turbid fluid already mentioned."

On this subject Dr. Latham has not, perhaps, been so clear as on others, but he mentions that the stools sometimes consisted of "some colourless turbid fluid." It is evident, therefore, that he saw the colourless turbid fluid, although he does not, I think, take any notice of the "deposit." It is to be remembered that the Doctor was describing a disease that was new to him, and that many who have written on cholera have taken no notice of this argillaceous deposit. Add to this that he has stated that "there was every degree and species of flux that was ever seen or described from the cholera of India," &c.

I think I have now pretty clearly pointed out the affinity of the two diseases, without at all drawing upon the resources derivable from my own experience of either; and I wish it to be understood that though I have, for obvious reasons, preferred to derive my arguments from the "account of the diseases of the Penitentiary," it is not by any comparison of that account with the decription of the present epidemic, that I have come to the foregoing conclusions, but from my own knowledge of cholera and of scurvy in various parts of the world. I trust I may now, therefore, be permitted to observe that I have repeatedly seen this argillaceous deposit, resembling chalk and ground spermaceti moistened with dirty water, in the stools of persons with scorbutic dysentery or diarrhœa. In the very last case of the autopsy of scorbutic dysentery which I witnessed, on the rectum being removed for facility of investigation, a considerable quantity of liquid escaped from it resembling curdled buttermilk, or the semi-fluid part which is sometimes observed on opening a new-laid egg, but less white.

Possessing due confidence in the general correctness of these views, it is my intention to write them out in such an extended form as may render them fit to be submitted to the consideration of the profession, but I considered it was my duty to lay them in the first instance before the Board of Health, even in this crude shape, and without delay.

I have the honour to be, Sir,
Your most obedient servant,
CHARLES CAMERON,
*Surgeon, R. N.*

*W. Maclean, Esq. Secretary, &c.*

*Central Board of Health, Whitehall.*

---

III.

### MR. DOBSON ON THE EPIDEMIC CHOLERA OF LEEDS, 1825.

I HAVE been induced to submit to the public, the few following observations on a disease which prevailed in the North of England, during the summer and autumn of 1825; in consequence of the remarks which were made by Doctor Johnson at the last meeting of the Westminster Medical Society, relative to the subject; I should not have obtruded any remarks of mine, but from the conviction that they might assist in elucidating, more clearly, the bearings of this momentous question; which is so much occupying the attention of the profession and the public; and having had a very considerable share of the cases, *at that period*, under my own observation; more especially of those which occurred to an alarming extent in a small village named '*Gawthorpe.*'

The facts which I shall present may tend to show how far the disease which prevailed in 1825, resembles the one which now exists in London and its vicinity. I need merely mention, *in limine*, that the 'common English Cholera,' always prevails to a greater or less extent during every Summer; but few cases terminate fatally, and these only amongst the aged or debilitated. But in the Summer and Autumn of 1825, the cholera presented so many entirely new features, such anomalous and unaccountable symptoms, that the medical men were alarmed at the aggression of so appalling a disease. Nevertheless, as the disease progressed, the alarm, in a great measure, subsided. The most energetic measures were adopted, not to arrest the progress of its ravages, but to relieve the anguish of the sufferers. Prophylactic measures were a secondary consideration. 'Sufficient for the day was the evil thereof.' Every day new victims were immolated, or suffered from its ravages. Day after day, new cases presented themselves; but no one knew why or wherefore. Some considered it contagious, and secluded themselves; others did not fear. Friend visited friend, and neighbour assisted neighbour, with assiduous attention. Human interest gave way to human sympathy. It was not before the middle of Winter, that the disease disappeared; and when the means of subsistence, and health, re-occupied the cottages that had contained nothing but wretchedness and disease.

Language is inadequate to depict the distress which prevailed; for to conceive it, it should have been seen. The disease presented itself under various forms;—and perhaps the best arrangement that can be adopted, will be to consider it under the three divisions which it alternately represented, viz.—Cholera, Diarrhœa, and Dysentery. But it seems imperative, previously to doing so, to make a few cursory allusions to the season of 1825; the nature of the locality where the disease occurred; and the characters of the residents. These I shall quote from the very excellent pamphlet recently published by my excellent friend and former preceptor, Mr. Thackrah, of Leeds. "In May, 1825, 3-8ths more rain fell than the usual quantity; and the wind was east nearly the whole month. In June, the weather was showery, and, on some days, particularly sultry; on the 12th, the thermometer reached 87 degrees; the wind was variable; but principally west. In July the weather was sultry, barometer high, winds variable, and the quantity of rain particularly small, not exceeding 1-5th of the usual quantity. About the 3rd of August, the drought which had prevailed for several weeks, was relieved by refreshing showers: during this month the quantity of rain exceeded the average, the weather was sultry, the barometer low, and wind generally west."—p. 24-25.

"*Gawthorpe*, four miles west of Wakefield, on high ground, and almost destitute of wood, is inhabited chiefly by weavers, who, though not generally poor, are dirty in their persons, and have their houses more than commonly filthy, and ill-ventilated. Few, if any, persons above the lowest class live in the village. As there is no public well, the water drunk is obtained chiefly from ponds. 'Of 70 houses which we examined seriatim, only 17 had been exempt from cholera and dysentery.'"

The above refers, I believe, to the month of September, when I accompanied Mr. Thackrah to see the patients. They were, at that time, about 30 in number. Not one house, when the disease ceased had been exempt, as far as I recollect.

In May and June, cholera appeared under its usual form. A great number of individuals were attacked, but few died. The symptoms were such as appeared in the common cholera, therefore need not be specified. But in July and August the disease assumed a more aggravated character. The mode of attack, and the progress of the disease, differed from the preceding cases, and the result was more fatal.

A person would be suddenly attacked at work, or on returning to his home, or in bed, with pain in the abdomen, particularly around the umbilicus, purging almost immediately ensued, first, of common feculent matter, but the intestinal contents being soon evacuated, then followed watery, or mucous stools, with excruciating tormina before each evacuation, continual and urgent calls to stool, and painful tenesmus. If appropriate remedies were at this stage promptly applied relief was generally obtained. Some cases, however, baffled every endeavour to even mitigate them; while others admitted of temporary relief; but soon the disease would re-appear, and with augmented vigour. *The most alarming prostration of strength would come on, with vomiting and purging of large quantities of fluid, resembling gruel or barley water, occasionally of the colour and appearance of a solution of mashed peas: cramps of the legs, and sometimes of the arms; intense spasmodic contractions of the abdominal muscles: pulse quick, and often imperceptible, skin cold and clammy, sometimes confined to the extremities, at others extending over the whole surface, countenance vacant, face of a most ghastly aspect, eyes sunk in the orbits and motionless, the eyelids and around of a purple, or more correctly of a leaden hue, lips purple, the face, even of a young and plump individual, became contracted like to the face of a toothless old man in a few hours: the patient would lie on his back with the extremities extended, the arms lying by his side, the skin of the fingers corrugated and the cellular tissue beneath exceedingly dense, in short it was like the hand of the dead: respiration slow, labouring, and imperfect.* Brandy, opium, ammonia, and every stimulant that could be suggested, in conjunction with artificial heat and friction over the body would fail to resuscitate. *In the course of a day, or often of a few hours, death would result.*

*In other cases the complaint would suddenly appear in all its malignancy, without any premonitory symptoms. The patient would sink in two or three hours under accumulated agonies, without even the slightest re-action taking place.*

During the autumnal months the disease still prevailed, but it was now almost invariably ushered in by prostration of strength, severe diarrhœa, and typhoid fever; these generally existed for a few days before the more urgent symptoms appeared; vomiting only when fluid was taken, or at least seldom otherwise, and then only what had been previously swallowed. The intestinal secretion changed its character, from a gruelly fluid to a soft gelatinous mass, or to mucus streaked with blood; sometimes it was like the washings of meat, large flakes of lymph floating in the liquid; frequently scybalæ of the size of a walnut. Previous to dissolution discharges of blood took place from the bowels to a considerable amount. The amount of discharge from the bowels, of whatever kind, was almost incredible. *The most prominent typhoid symptoms marked the disease throughout its continuance. Death in general ensued on the fourth or fifth day.*

The treatment of cholera consisted of the application of heat to the surface, brandy and ammonia, and opium in the dose of two and three grains in a pill. Tincture of opium could seldom be retained on the stomach, nor even brandy, in many cases. One circumstance I find noted in my manuscripts, and one which was always observed, that fluids constantly provoked vomiting, and therefore were strictly prohibited in *every case*: considering them as irritants, which when applied to an already irritable and excited organ necessarily *increased* its irritability.

### Diarrhœa.

The next consideration is the diarrhœa which existed *unconnected* with cholera.

Diarrhœa during this season was very prevalent; in short, few individuals who resided in 'Gawthorpe,' or who visited the sick there, were exempt from it. It arose frequently without any obvious or assignable cause; occasionally it was traceable to indigestible food.

Frequent purging; griping pains in the bowels, especially before each evacuation: the characters of the dejected matters were analogous to those observed in the cholera cases. Extreme debility; typhoid fever; cold extremities; rigors; tenesmus. Relief could sometimes be obtained in a few hours, especially if aid was had soon after the occurrence of the attack; but if deferred for a few days, the complaint would continue for weeks, or even months; and resist every means. Three individuals in one family, and many others, had diarrhœa for months afterwards; particularly when they had hot food or drinks, which used to pass the bowels unchanged: these, during the progress of acute cases used to be prohibited, in consequence of the irritation they induced in the bowels.

*Diarrhœa,* though not so distressing to the patients as cholera, caused in the minds of friends considerable alarm; and knowing it to be so common a precursor of cholera, medical aid used to be procured as early as possible after the commencement of the attack. Not many deaths ensued in this form of the disease.

The remedies for diarrhœa were simple and generally efficient; 3 or 4 grains of calomel with one or two of opium, in a pill, were given every four hours, until the spasms and the purging ceased; followed by a dose of oleum ricini, or tinct. rhei. If the disorder relapsed recourse was again had to the calomel and opium, conjoined, perhaps, with an aromatic cretaceous mixture.

### Dysentery.

Many of the cases of diarrhœa which, when continued beyond the fourth or fifth day; especially when medicine had not been taken, resolved themselves into severe cholera or dysentery; when the latter, there was an enormous discharge of blood from the bowels, pints, nay quarts in a day were discharged; sometimes pure uncoagulated blood, at others commixed with a known watery fluid, sometimes with a fluid like the washings of meat. Scybala of a large size were often passed. Most distressing tenesmus. Pain at the transverse part of the arch and the sigmoid flexure of the colon. Typhoid fever of a most *malignant* character. Delirium; suffused eyes; headache; brown parched tongue; sordes on the teeth; quick pulse; involuntary discharge from the bowels; retention of urine. In one case, that of a woman about 30 years of age, the most distinct petechiæ and buboes appeared a few hours previous to death. A large majority of those cases terminated fatally.

Dysentery required the most energetic treatment. Six or ten grains of calomel, with two or three of opium, were exhibited every two or three hours until mercurial action was fully established: when the mouth could be freely affected the disease began immediately to subside, and recovery took place. This is, I conceive, a very valuable practical fact; unfortunately it was not until the latter period of the epidemic that this practice was enforced—when experience, negative knowledge, had proved the inefficacy of small doses of medicine. General bleeding was seldom resorted to; never unless there were marks of decided peritoneal inflammation: only once or twice, I believe, was the lancet used. Leeches at the seat of pain, and hot fomentations were of service. A ball of solid opium placed in the rectum relieved the tenesmus. For the head affection, blisters, &c. But after every effort and attention death frequently resulted. The disease would appear arrested, but after a short respite, would come on with increased vigour. *Ten corpses* lay in the village at the same time.

I may mention, that in Gawthorpe there were a few cases of typhus fever, without vomiting or purging. In some of the *fatal* cases, however, diarrhœa preceded, and hastened death.

Should these few cursory observations afford any interest to the members of the Westminster Medical Society, the author will be amply repaid.

W. DOBSON.

9, *Belgrave Street, South, Pimlico.*

We beg to draw the particular attention of our brethren to this most important document, which, in connexion with the extracts from Frank, and the observations of Dr. Brown, of Sunderland, contained in our last number, will clearly shew the intimate relation that subsists between fever and epidemic cholera, so called.

If any one can doubt, after the papers presented in this number of our Journal, that diseases greatly analogous to that now epidemic in the metropolis, did occur in England previously to 1831, he must be very sceptical indeed. As doubts have been thrown on the accuracy of Mr. Thackrah's delineation of the Leeds epidemic, as seen in our Review department, we are happy to give the testimony of Mr. Dobson, as to the scrupulous fidelity of Mr. T.'s narration. *Ed.*

---

### IV.

OBSERVATIONS ON THE HISTORY AND TREATMENT OF CHOLERA ASPHYXIA, AS IT HAS APPEARED AT HADDINGTON. By ROBERT LORIMER, M.D. and JOHN BURTON, M.D. Secretary to the Medical Boad, 8vo. pp. 64, Stitched. Edinburgh, 1832. Price Two Shillings.

PAMPHLETS ON Cholera are crowding upon us, and nothing but Cholera is read of, talked of, written of, or dreamt of in the medical world. Our readers will find a very valuable body of evidence and opinion on the subject in the present number of this Journal, and we hope, if the pestilence spares ourselves, to exhibit a complete picture of its progress and character in this country. We have this month shewn it as it appeared in the Presidency of Madras, in Hindostan, and portrayed its features at Sunderland, Newcastle, Gateshead, Hillhead, and Musselburgh. We are now to display it at Haddington.

On referring to Brookes's Gazetteer, we find that this Borough is sixteen miles East of Edinburgh. Its population was 4,370 in 1811, and 5,255 in 1821, an increase of nearly a thousand in ten years. Supposing that the increase has proceeded at the same rate, and it ought to advance at a greater, the population at present will amount to upwards of 6,000. The cholera commenced in the place on the 17th December, 1831, and on the 23rd of February 1832, the total number of cases was 125, or 1 in 48; the total of deaths 54 or 1 1/9 in 111. Of the 54 persons who died, 22 were men, 30 women, and 2 girls. Of the 125 who were attacked, 50 were men, 66 women, 1 boy, 8 girls. The female sex has hitherto suffered much more than the male in this country.

We are pressed for space at this late period of the quarter, and must confine ourselves strictly as possible to the enumeration of facts. They will speak for themselves.

"The town of Haddington is situated on a plain about a quarter of a mile square, bounded on the south and east by the river Tyne, having its course eastward.

The elevation of the town above the level of the sea is somewhat under 100 feet, and the whole surrounding country, except on the river-side, rises from it as a centre. The streets in the town are wide and airy, but there are numerous sections by lanes, which are ill-ventilated and filthy.

The suburb of Nungate, irregularly built, also abundantly filthy, ill-ventilated, and inhabited principally by the poorer classes, is on the east side of the river, (about one-hundred yards wide here,) and the line of communication is by means of a bridge, the water being pent up by a milldam running across from that part of the town in which the disease first appeared, to the Nungate side. It is worthy of remark, that the refuse of three tanworks, the town slaughter-houses, and two common sewers, intersecting the town, is discharged into the river at or near this place.

The Cholera made its first appearance in Haddington on the 17th December, 1831; and, after a few straggling cases, between which, with one exception, there was no communication traceable, it became general; confining itself, however, more particularly to the eastern districts of the town, and to the suburb of Nungate.

The first patient was a confirmed drunkard, and had been exceeding in the use of ardent spirits more particularly for the two days preceding his attack. His residence was in a steep narrow lane, leading down to the river, in the vicinity of the slaughter-houses, and forming one of the avenues to a tan-yard and bone-mill, where there was a large depôt of bones, &c. gathered from the surrounding country, and not imported from Hamburgh, as was reported at the time. If filth, dissipation, and the combined influence of many causes acknowledged to have a direct effect in depressing the vis vitæ, can, without the agency of contagion in its true sense, produce Cholera Asphyxia, we think it will be allowed that they had full scope for exercising their power in this case; and, *as there was not the most distant probability of the man having come in contact with any one labouring under the disease, or from the infected districts, we may certainly be justified in concluding, that the first case in Haddington was sporadic.* The assertion that the disease was 'brought from Newcastle,' is too vague to have deserved notice, were it not that, through the public press at the time, it was asserted, with much confidence, that three shoemakers were the unhappy medium of conveying this malady. If this were the case, the men must consider themselves more than fortunate in having escaped the addresses of their unsparing fellow traveller. The truth is, the three persons alluded to, named Frazer, Gow, and Walker, left Newcastle, on the 9th or 10th of December, travelling on foot, resting eight nights on the road, at different stages, and arriving in Haddington *after the first case had been ill for twenty hours.* They never saw the man, they had never seen a case in Newcastle, have never been attacked themselves, nor have they communicated the disease to any of their families or neighbours."

Having seen how the disease commenced it becomes necessary to inquire next how it subsequently extended.

"As already remarked, the first case appeared on the 17th December; the second (*on the 25th*) a girl, æt. ten or twelve, of a delicate constitution, who lived in the neighbourhood of the first. *She had no communication with him, or the inmates of the house in which he resided, but had been exposed for some time in a thin dress, on the evening before her attack, which proved fatal in a few hours.* The house in which she lived communicated with the entry to the tan-yard, was damp, ill ventilated, and inhabited by her father, mother, and numerous family, all of whom were more or less exposed to the influence of contagion, had it existed; yet not one of them had the disease until the 3d of February, when the eldest daughter, æt. 18 (who, from her occupation as a straw-hat plaiter, was necessarily much from home in another part of the town), was attacked, and fell a victim to it. The family were thus again exposed to the risk of infection; but, up to this date, remain well, although no separation of the healthy from the sick was practicable from the limited extent of their dwelling, and no means of prevention have been used except whitewashing the house and washing the bed-clothes.

The third and fourth cases were women living in the same tenement, but in different apartments. Their house is situated in one of the dirtiest closes in the town (leading also to the river), about 150 yards from the residence of the last case. They both fell victims to the disease, without communicating it to any of the numerous visitors led by curiosity to see them during their illness, and after death. The husband and niece of one of the women removed to the family of a brother-in-law, *who was much engaged with them in attending their relation, and in cleaning the house, &c after her death.* Four or five days after being thus exposed to the influence of all the causes producing the disease in his wife and her neighbour, the husband was attacked in the house of his brother-in-law; and, on the same day, the niece and brother-in-law, *who also were exposed to the same causes in the poisoned locality.* The husband perished in six hours and a half; the niece and brother-in-law recovered; the girl, after a dangerous illness of three weeks. Here, let it be remarked, that none of the brother-in-law's family, in which they were ill, or the families in the tenement in which they lived, have been attacked, although nothing whatever has been done towards fumigating or whitewashing the house.* Owing to the long illness of the girl, it was not practicable to remove her for that purpose, and, since, it has been overlooked.†

Isabella Macleish, mother of case first in Appendix, died in a house in the Nungate, on the opposite side of the river from the close in which the third and fourth cases died. *She was a most dissolute character, and had no communication with any of the previous cases.* Three other women of similar habits, living in the same tenement, were subsequently attacked and died. Macleish's two children, and a child of one of the other women, had also symptoms of the disease; but none of the other inhabitants of the tenement have been attacked. One of these is an old man, with cancer in the throat, and a debilitated constitution, living in the story immediately above the rooms of Macleish and the others.

George Patterson and his daughter, also residing in the Nungate, though at some distance from these cases, took the disease on the same day: he died of it, the girl recovered; *but none of the family (though they all slept in the same room) have been attacked.*

Mrs. Robertson, also in the Nungate, on the outskirts of the suburb, died of cholera, and, though she was surrounded by a numerous family, none of them have taken the disease. Many similar instances could be adduced among the lower classes. Let us now, however, view the course of the disease among the more respectable inhabitants of the town.

After a remission of the disease for nearly eight days (which took place upon the return of soft weather), we had a renewal of it (with great violence during two or three days) following a gale of wind from the north and east, with rain and snow. In this attack the better classes were its principal victims. In their families, the disease has been confined entirely to those first attacked, they being predisposed to it in almost every instance, and contagion here is altogether out of the question.

One of the first of these cases was that of Mr. S. who had been subject to disorders of the stomach and bowels for some years. He died in the midst of a numerous family. Mr. A. also fell a victim to the disease, surrounded by his family. Mr. M. was attended by his mother, sister, and servant. Mrs. A. and

---

* "For three days after the husband's death all his bed-clothes remained in a heap at the side of the bed, *to which the niece was removed immediately after her uncle's death;* and in the same room (ten feet square) a family of eight or ten individuals resided."

† "A case occurred in the tenement on the 17th February."

Miss C. also died of cholera, and in none of their families have there been any farther spreading of the malady. So much for 'contagion' in this town. Its influence has not been more perceptible in the country, as far as we have had access to it.

The case at Beanston Mill was truly a sporadic case, the subject of the attack being a poor woman who had neither been in Haddington, nor had any communication with any person from that place since harvest. She died of the disease after reaction had commenced. During her illness, she was well attended by several of her neighbours, besides receiving casual visits from many more. Not one of them has taken the disease except her mother; this case being in its results a farther proof of the limited influence of contagion, although at first sight it appears like a confirmation of its virulence. The woman, after a residence in the house of her daughter for about forty hours, returned home to the village of Athelstoneford, about two miles off, and was attacked with cholera on the second day after her return. She had visited the poisoned spot and received the disease (when attending a patient?) she did not communicate it to any one in Athelstoneford, which she probably would have done had it been contagious. There was another case in the village at the same time, between which and the old woman there was no communication. None of the attendants of either have been since attacked.

The case at Knowes, six miles east of Haddington, was similarly a sporadic case. It is rather remarkable that both these cases are on the banks of the Tyne, and the general situation of both resemble that of Haddington. The poor woman who was the subject of the attack lost her life, but none of her neighbours, or her attendants, have been seized.

There was one case at the village of Whittinghame, in which the disease did not spread, and two cases at Bees' Knowes (about two miles distant from the latter), which had no intercourse with each other, although the other inhabitants of the hamlet had free communication with both, with impunity. At Ruchlaw Mill, also in that neighbourhood, there was a fatal case, which was not interred for three days after death. During his illness, and until he was interred, some of his family and friends sat up night after night in the house, and have not been attacked.

A young man, named Hardy, who had been working for a week in Tranent during the prevalence of the disease, to its greatest extent in that place, came to his home at the village of Gladsmuir, and took the disease, but did not communicate it to his family, who are known to be highly predisposed.

A woman, who was afterwards the subject of our first galvanic experiment, left Tranent while labouring under an attack of diarrhœa, travelled on foot to the village of Samuelstone, became collapsed there, and was removed in a cart to Haddington. None of the people who saw her, and assisted in removing her, have taken the disease.

Two of the servants of a gentleman's family, about seven miles east of this, took the disease, having had no previous communication with any of the infected persons or districts; they both recovered, and the disease has not spread in the family nor neighbourhood. There was also one case at the village of Whitekirk, in which the disease has not spread.

The disease has not attacked the village of Linton, five miles east of this, although the inhabitants are in daily communication with all the above places. It prevails at West Barns, four miles farther east. It has not attacked Belhaven nor Dunbar, both within two miles of West Barns."

The natural comments on, and corollaries of the foregoing facts, will suggest themselves to the minds of men of reflection, habituated to exact reasoning. We have not space for any.

"The disease, though it be admitted to have travelled down the river (as has been alleged), has not as yet ascended its course, nor have any of the inhabitants of high grounds around the infected localities been its subjects.

It is remarkable, that, in its progress, very few instances of the disease have occurred in persons perfectly healthy; they, to a certain extent, appearing to enjoy something like immunity, from the severer symptoms at least; the aged poor and laborious, the dissipated, with those subject to disease in the digestive organs, and whose minds were weakly constituted, being among its earliest victims, and almost invariably experiencing the severest attacks."

The management of the preliminary stage of diarrhœa was generally easy, but almost all the patients seen for the first time during the stage of collapse perished. This, we fear, is too generally the case.

"The modifications produced in this disease by atmospheric changes open a wide field of enquiry, and we are sorry that our limited opportunity does not enable us to say much on this head. The accession of the disease here was during a hard frost, with northerly and westerly winds; and, on the other hand, mild weather, with south and west winds, prevailed upon the first remission;—increasing tenfold with severe weather and north-east winds, and now again decreasing with south and south-west and frosty nights.

The diet of the poorer classes in this town and neighbourhood consists principally of vegetable matter, and the use of ardent spirits is more or less general in the same class. Nearly all the medical attendants here were more or less affected with a peculiar uneasy feeling in the abdomen, sometimes amounting to tolerably sharp pain, and these feelings seemed to be increased, after an attendance upon the patients for any length of time in their own dirty and ill ventilated dwellings.

The disease among the poor has decreased both with regard to the number of cases, and the severity of the symptoms, since the establishment of a soup-kitchen, and clothing depôt. We mention this as a fact, without, of course, pledging ourselves to account for it."

The most common premonitory symptom was diarrhœa, varying in duration from a few hours to several days or even weeks, before the more violent attack. The following proved the most successful treatment.

"Diarrhœa with or without griping pain in the bowels, often yielded to a dose of castor-oil, or one or two mild doses of rhubarb, magnesia, and ginger, with rest and warmth, and a duly regulated diet. In some cases, an opiate preceded the exhibition of the aperient, and with decided advantage. When the diarrhœa was attended with frequency of pulse, heat of skin, and even a certain amount of collapse in the features, shewing the livid areola about the eyes, a small bleeding has been often practised; and as an effect, the patient generally says he 'feels lighter,' though not previously sensible of any particular depression. One grain of opium, with three or four of calomel, repeated in a few hours, according to circumstances, was next administered, and followed up, after a proper interval, with castor-oil, or divided doses of the compound extract of colocynth with rhubarb.

The purgatives required repetition for several days, till the alvine evacuations, thinner and darker than natural (sometimes pitchy), regained their usual appearance. The diet was of course mild and moderate in quantity, and the patient kept constantly in bed."

Galvanism was tried in several cases during the stage of collapse. In all but one some sensible effect was produced.

"The results might have been more satisfactory than they were in cases 21 and 22, if a battery had been used instead of a single trough of *sixty* plates; and if the trough itself had been in a state of more perfect order. But, so far as the results go, it appears reasonable to conclude, that, with a more perfect and powerful apparatus, and more vigorously constituted subjects than those operated upon, a greater and more permanent benefit might be obtained."

Twenty-three cases are related, the last a very curious one as illustrative of the effects of the galvanic power. Our limits, however, compel us to stop. We think the able authors of the present pamphlet deserve great credit for coming forward as they have done. Let the chief practitioners of every town or district where the cholera prevails, investigate with the utmost care the cases that they witness, and when the pestilence is gone by, let the results be published for the instruction and benefit of the community. We do not recommend every surgeon and physician to write a pamphlet. God forbid. Perhaps the best means of arranging and finally of giving to the world the results of their observation and experience, would be for the medical practitioners of the place where cholera springs up to form an association between themselves. This association should constitute the focus to which all individual reports should be directed, and from which a joint report should ultimately spring. The whole should be on the model of the Indian reports, the best that have ever appeared upon any epidemic. We would state most explicitly that the association should not be made a job; it should be open to each and all.

---

### V.

### History of the Progress of Cholera (Choleric Fever) in England.

With the view of putting on record something like a connected view of the history of the epidemic in England, we have committed that task to the hands of a gentleman well qualified to perform it. But as we have left him at liberty to embrace any tenets or doctrines which he pleases, so we hold ourselves entirely free from responsibility on any points of speculative reasoning which may be broached in this history.—Ed.

---

During the latter end of the Summer and Autumn of last year, bowel complaints under the form of diarrhœa, and cholera, had been very prevalent at many places throughout this kingdom. The very great severity of many of the cases of cholera, an unusual number of which proved fatal in the short period of 38, 24, or even 18 hours and less; gave rise to alarms that the scourge which had been devastating the Continent of Europe had appeared in this country. At Port Glasgow, Leith, Hull, and Margate, these reports were successively raised; whilst in many other places, the existence of the same diseases was passed by unnoticed. Such was the case in Sunderland and its neighbourhood, where this epidemic first appeared in all the continued malignity and extensive prevalence by which its progress in other countries has been marked.

Sunderland.—On the 26th October, a man of the name of Sproat who resided at Long Bank in Sunderland, died of the cholera:—he was about 60 years of age, a labourer, and had suffered under slight diarrhœa for several days before his death. About half an hour after the death of this man, his grand-daughter was taken ill, and the next day her father; both were removed to the hospital, where the girl recovered, and the father died on the 31st. On the same day also died, a keelman of the name of Wilson in High-street, and a shoemaker named Rottenburg, who lived in a low and confined house on the Monk Wearmouth Shore.

On the 1st of November another death occurred, that of a nurse at the infirmary; who had assisted in removing the dead body of the younger Sproat from the ward of the hospital, but who had not attended him during his illness. Subsequently to her having assisted in carrying the dead body, she was seized with extreme alarm respecting cholera, and the danger of being infected by the dead body. She died in six hours after the first attack. From the first to the 5th of November, no other case was noticed. On the latter day one case occurred; and on the 6th and three following days, the disease became very prevalent, 26 persons were seized, 14 of whom died. It continued to rage in Sunderland, with fluctuations in the degree of its severity; declining for a few days, and then again, for a like period, increasing until the third week in December; when the number of fresh cases diminished to rather less than one third of the previous average: from the 26th to the 30th, but five new cases are reported, and from that time the disease may be considered as having nearly ceased in this town. Up to the 24th January, but six new cases having occurred at distant intervals; this town then remained free from any case of sufficient severity to be reported cholera until the 8th of March. On that day two new cases occurred. The total number of cases reported up to this time are 538 and 207 deaths.

We have to remark, that the number of cases and deaths, has not been accurately reported; especially in the early part of the time. It became a matter of interest to the inhabitants, to conceal the presence of the disease amongst them, from the injury their commerce received by the quarantine regulations imposed under the belief that it was contagious, and to such a pitch did this public feeling rise at one time, that the reporters of the first appearance of the disease, were in danger of personal violence from the mob, if their names could have been ascertained; and many surgeons to ingratiate themselves with their townsmen, were charged with concealing and misrepresenting the nature of the cases which occurred.

Up to Nov. 10, the severest cases only were reported. From the 11th to the 21st, they attempted to conceal the nature of some of them under the name of common cholera, and even of diarrhœa; but after the 21st, the column for diarrhœa was discontinued, and after the 26th, that for common cholera also. All those cases of the diseases of the season, which did not greatly surpass the ordinary severity of diarrhœa being then altogether excluded. It is greatly to be regretted that some correct classification of the cases according to their severity, was not subsequently attempted: for a knowledge of the proportions which the different forms of these disorders of the alimentary canal bore to each other, together with the observation of the circumstances under which each occurred, would in a great measure have enabled as to ascertain the causes of the disease.

Nevertheless, that cases of diarrhœa were more prevalent than those assuming the milder or severer forms of cholera, we learn from the comparison of the mortality during these 11 days with that of all other subsequent periods. Thus from the 11th to the 21st of November, of cases under the head of common and malignant cholera, we have 85 new cases, and 33 deaths; and under the head of diarrhœa, 111 new cases and 4 deaths; for the first period of 11 days after the 21st of November, 126 new cases, 49 deaths; for the second, 105 new, 51 deaths, and for the third, 73 new cases, 39 deaths. Now taking the mortality of the first period at one third, being the lowest proportion reported, and adding 12 of the diarrhœa cases to the column of cholera, as all that could have been erroneously reported, the proportions will be 97 cholera, 99 diarrhœa, the latter still preponderating.

We extract the following interesting observation, on the Statistics of Sunderland, from the work of Mr. Parsons.

"The town of Sunderland consists of three parishes; viz. Monk Wearmouth, with a population of about 6,000,—Bishop Wearmouth, with 14,000,—and Sunderland with 20,000 inhabitants. In Bishop Wearmouth nearly all the wealthy inhabitants of the town reside, and the number of its pauper population is very small; the streets are generally sufficiently wide and clean, and their elevation above the level of the River is from 110 to 120 feet. In Sunderland, which is a continuation of Bishop Wearmouth, there are very few streets of proper width, and their general elevation above the level of the River is from 70 to 90 feet lower than in the adjoining parish. The bye-streets are extremely narrow, several not being broad enough for the passage of a common cart, and during my residence in the town they were rarely cleansed from the dirt, and other impurities allowed to accumulate in them, for many days together. The houses in these bye-streets or lanes commonly had no yards or courts attached to them; the rooms were dark, ill ventilated and dirty; the passages and stairs were dirty through the great number of persons living in each house; and very often each room, from the cellars to the attics, was occupied by a whole family:—Dr. Barry found 120 individuals living in one of these houses. In the parts bordering on the River, Monk Wearmouth closely resembles Sunderland in the crowded state of its poor inhabitants. For several years past it has been the custom in Sunderland for the parish managers to contract with some individual for the maintenance, &c. of the whole poor of the parish. When this plan was first adopted, now about seven years since, the annual expence for this purpose amounted to upwards of £9000, the number of persons contributing to the poor-rates being at that time, as it is at present, rather more than five hundred, and consisting almost entirely of the shop-keepers and others necessarily resident in the place; but, by means of the farming system, the annual expence has been gradually reduced to its amount in the present year, of between three and four thousand pounds, with, it is believed, a considerably increased number of claimants on the parochial funds. A necessary consequence of this cruel economy, was the state of great destitution in which the poor existed at the time of the appearance of Cholera amongst them; and, although much was done towards improving their condition, by giving them clothes, bedding, fuel, &c. still it was found that individual exertions were manifestly unequal to counteract the ill effects, on the public health, of extreme and widely diffused poverty, and its too frequent consequences, a neglect of cleanliness, drunkenness from the immoderate use of ardent spirits, and the gratification of other depraved propensities. The death, by Cholera, of Mr. Middlebrook, the late contractor for the poor, has released the parish from its engagement to him, and the system of farming the paupers is now, though late, abolished." 10.

The ravages of the disease were chiefly confined to the parish of Sunderland; in Bishop Wearmouth, which contains at least one-fifth of the whole population, but only a small portion of the pauper inhabitants of the town, a few cases occurred in the middle of November, and again in the beginning of December; but up to the 12th of that month the number of deaths in this parish did not exceed 20. In the parish of Monk Wearmouth but few cases occurred, not more than 15 deaths having taken place from the disease up to the same date.

With regard to the parish of Sunderland itself, the disease was almost exclusively confined to the low, dirty, and confined lanes in thickly populated districts, not more than 12 cases having occurred in the upper and more widely-built portion, although the freest and most unrestrained communication existed with those places where the disease raged.

It was strangely at variance with all preconceived ideas on the subject, that this disease should remain so long confined to Sunderland, although restrictions on the inland intercourse, between the inhabitants of the town and surrounding country, were not imposed; and those who had expected that its progress there would be in proportion to the rapidity of communication between the different towns of this country, began to entertain the delusive hope that its ravages would not extend further.

Although one case had appeared at Newcastle as early as the 26th October, and five between the 28th November and 9th December, it did not prevail extensively here until after the 10th of this month, and about the same time it broke out in various parts of the surrounding country. In the neighbourhood of Newcastle, Walker Township and Colliery were now affected, together with Sedghill, New York, and Wideopen Collieries. Along the Coast it appeared at Blyth about seventeen, and at North Shields about seven miles north of Sunderland, and at Seaham, a small fishing port about four miles south. There were rumours of its having occurred also at Alnwick and Chester-le-Street, although not noticed in the official reports. It also appeared about this time in the extensive parish of Houghton Le Spring, on the south bank of the Wear, between Sunderland and Durham, and on the 17th December at Haddington, in North Britain, 105 miles distant from Newcastle. It now becomes a matter of extreme difficulty to trace its subsequent progress with any degree of accuracy, owing to the irregular manner in which it spread over the country, and still greater irregularity with which its appearance at most places is reported.

Whilst extending along the south shores of the Frith of Forth from West Barns to Edinburgh, it also continued to affect in the most capricious manner various places in the neighbourhood of Sunderland and Newcastle. On the 22d of January, it appeared at Kirkintilloch, on the Forth and Clyde canal, about seven miles from Glasgow, and in the beginning of February it broke out in London, in Glasgow, and various parts of that very populous neighbourhood, still continuing to extend around the places which had been the theatres of its former irruptions.

From this extreme irregularity of the progress of the disease, it becomes very inconvenient to notice the places attacked in the order of time when it first appeared in each. Our narrative will be less broken by arranging them in the order of proximity to the place first affected.

SEAHAM.—Along the eastern coast it has appeared at the fishing port of Seaham, about four miles to the south of Sunderland. It does not appear to have prevailed very extensively in this place; for, from the 15th of December, the date of the first, to the 21st, the date of the last report, only seven cases are noticed, five of which were fatal.

NORTH SHIELDS AND TYNEMOUTH.—This place lies about seven miles to the North of Sunderland, at the mouth of the Tyne: it is remarkable for the long time during which the cholera has continued to prevail, and the slow progress which it has made; from the 11th December to the end of the month, only 19 cases are reported, and these occurred on the 11th, 13th, 19th, 20th, 21st, 22d, 23d, 27th, and 30th.

In January cases began to occur every day; in the first week 20, in the second 24, in the third 23, in the fourth 45, and in the fortnight from the 29th January to 11th February, 122. We ought to observe that in the latter part of this time are included cases from the neighbouring townships of Murton and Chirton. Up to the 3d of March 334 cases and 97 deaths had occurred.

SOUTH SHIELDS.—At this place, which lies on the opposite bank of the Tyne, nearer Sunderland, it is remarkable that the cholera did not break out until the 27th December, and then prevailed only to a very trifling extent, eight cases having occurred and three deaths only in the first four weeks after its first appearance, and in this number is included cases from the neighbouring townships of Westoe and Hebburn colliery.

It has also appeared along the coast at Seaton Sluice, a few miles to the North of Tynemouth; at Blyth, ten miles distant, and at Hartly, where are extensive collieries, glassworks, and other manufactories, about four miles E.N.E. of Blyth. It is remarkable that its appearance at Seaham was in December, and this place was entirely free, when Hartly, close by, was severely affected in the beginning of February.

Next, following the course of the Tyne, it has been very prevalent in the towns and collieries which are scattered along its banks, and at a distance of a few miles.

At Earsden colliery, about four miles to the north of Shields, it broke out in January: the first reports from this place are dated the 25th and 26th of that month. On the first day 32 cases and 4 deaths are reported; on the 26th, 10; on the 28th, 6. Eighteen other cases occurred up to the 6th of February, and four on the 7th, after which the reports are discontinued; making a total of 74 cases, only 8 of which were fatal. In this place we observe a very small mortality; and it is probable that the majority of the cases occurred in the form of diarrhœa, for whilst some report only the severest of the cholera cases, others include many if not the whole of the cases of diarrhœa which prevailed at the same time: hence we cannot correctly ascertain the severity of the epidemic in different places, except by comparison of the mortality with the whole population.

Percy Main, the next place in order of proximity to Shields, situate on the North bank of the Tyne between Newcastle and Shields, was one of the last places attacked. The disease broke out here in the beginning of February with great violence; in ten days 90 cases having occurred, and 12 deaths. It afterwards continued to prevail, but with diminished fatality.

At Howden Dock and Wallsend, almost close to the former place, it appeared in the beginning of January, but with little severity; up to the end of the month, when the reports ceased, but 32 cases and 12 deaths having occurred.

Along the South bank of the Tyne we have already noticed Westoe and Hebburn in the reports from South Shields. The latter place is in the parish of Jarrow, which also contains the Chappelry of Haworth. Here and in the village of Jarrow, and also in the parish of Washington the cholera has appeared.

At Newcastle we have already stated that one fatal case of cholera occurred on the 26th October, the day that the first acknowledged case occurred in Sunderland. No other fatal case was afterwards noticed until the 26th of November, when a man of the name of Jordan died. This man was a labourer, of temperate habits, and previous good health, residing in a small and dirty room in the New Road, the floor of which was somewhat below the level of the street. From the minutest enquiry it did not appear that this man had been in any way exposed to possible sources of infection. The alarm was now raised, and other cases were noticed; but up to the 8th of December only five cases had occurred in all; two of which were fatal. Up to the 12th, 12 cases and 3 deaths had been reported.

In the course of the next seven days, namely, to December 19th, it began to prevail extensively; 86 new cases and 34 deaths occurring. In the week ending the 2d of January it arrived at the height, 211 new cases and 56 deaths being reported; from this time it declined slowly; from the 2d to the 9th day the new cases and deaths were 159, 52; from the 7th to the 16th, 118, 38 : from the 16th to the 23d, 86, 29; from the 23d to the 30th, 65, 26; from the 30th January to 6th February, 32, 7; from the 6th to 13th, 8.

The population of Newcastle is 70,000. In the early part of this time its ravages were confined to the banks of the river, particularly in Sandgate and the lanes running from it; but in the beginning of January many cases occurred in the higher parts of the town, as in Westgate, Percy Street, and Prudhoe Street.

Gateshead. Gateshead, a populous borough, containing about 10,000 inhabitants, and separated from Newcastle only by the River Tyne, with which place the most constant intercourse exists, presents one of the most remarkable circumstances hitherto noticed in the progress of the cholera in England. It has been considered a fact establishing the contagious propagation of this disease, that it should sometimes first appear at that end of a town which lies nearest the place previously affected; but although the road from Sunderland to Newcastle lies through Gateshead, and the most frequent intercourse exists between all these places, and cholera had raged with great severity in Newcastle for more than a fortnight, up to the evening of the 25th of December only two cases had occurred, one on the 14th and the other on the 24th. On the morning of Christmas day this disease broke out with the greatest violence, and in the short space of three days 142 cases and 55 deaths had occurred. On the 26th, 39, 10; on the 27th, 59, 32; and on the 28th, 44, 13. In the course of the first week 250 cases and 71 deaths are reported; but its decline was almost as rapid as its inroad; in the second week, of new cases and deaths there were 95 and 42; in the third week, 30 and 15; and from this time up to the 2d February, when the disease ceased, only 20 cases and 12 deaths are reported. Seven of these appeared on the 7th of February.

In Gateshead, as in all other places, it prevailed to the greatest extent in particular parts of the town, chiefly in Hillgate, Pipewell-gate, Oakwell-gate, Jacsonk Street, and Bottle Bank. As usual, the chief number of its victims are found amongst the most wretched class of the inhabitants; but here a very considerable number of working people in more comfortable circumstances were affected, and its extraordinary violence during the first few days has been ascribed to the powerful assistance which gluttony and drunkenness have always been found to give to the other causes of this disease; and it is well known that Christmas Eve is devoted to the enjoyment of good cheer among the lower ranks in this country; and that drunkenness prevailed to a frightful extent in Gateshead on Christmas Day, we have the testimony of many witnesses. But it is another of the strange anomalies presented by this epidemic, that other places, as the lower part of Newcastle, were equally dissipated, and infinitely more exposed to contagion, situate also not many hundred yards distant; thus precluding the possibility of the existence of any different atmospheric state: and yet the irruption of this disease was simultaneous over many parts of the parish, in a track 3½ miles around the Borough. At Gateshead Fell, up to the 27th, 10 cases had occurred and 6 deaths; and it subsequently prevailed with much severity; but such reports as have been given are included in the former. This village occupies a high and bleak situation, about two miles from the Borough, and in this respect forms a striking contrast to the low confined parts of the towns of Sunderland and Newcastle, where the cholera has chiefly raged; but it appears to us that too much has been attributed to bad air and dirt: no doubt some part of the effect is due to them; but most is to be ascribed to the constitutions of the usual inhabitants of such places. At Gateshead Fell, the abodes of squalid poverty have been the domicile of this disease; and the houses in which it has occurred are said to have formed one of the most miserable features connected with this epidemic. The subjects of attack were seen lying in pairs in the same room with the dead, or those labouring under severe symptoms.

In many of the collieries and villages around Newcastle the cholera has prevailed to an alarming extent; in others it has been very slight, and some isolated cases have occurred in the inhabitants of lonely houses, far removed from all previous communication with the sick.

To the east of Gateshead Fells Ayrton Bank, has suffered; to the south some cases appeared at Chester-le-Street, in the early part of December, but it has not subsequently prevailed. In the beginning of January, Wickham was attacked a little to the east; and in February a case occurred at Friars Goose.

In Newburn, a village containing about 400 inhabitants, about five miles from Gateshead, the cholera commenced on the 1st of January. During the last half of that month it prevailed with very great severity, attacking upwards of 200 persons. In February it declined; and up to the 9th of this month the mortality had been 57 out of 300.

In this irruption the rector and some other persons of comparative opulence fell victims. The disease was not entirely confined to the village, but extended itself over a considerable portion of the parish, which contains upwards of 5,000 inhabitants, scattered over a space of about five miles.

In the villages and collieries to the north of the Tyne, around Newcastle, the cholera has prevailed with varying degrees of severity, breaking out and again disappearing in the most irregular manner.

To the West and N.-West, Dunstan, 2½ miles distant, was affected in the last week of December; subsequently it has prevailed extensively at Benwell, three miles distant; it appeared in the middle of January, and also in the neighbourhood of Elswick, Scotswood, Bells Close, and Leamington, ceasing about the end of January; but breaking out in Walbottle, New Wynning, Black Collerton, 4¼, and Wylan, nine miles distant, in the beginning of February. At Halswhistle, eighteen miles distant, one fatal case occurred. A young man, who had left Newcastle on a visit to some friends at that place, was taken ill with the cholera immediately on his arrival, and expired in a few hours.

To the N. and N.E. of Newcastle, Seghill, and New York, and Wide-open Colliery were affected, about the same time as Newcastle, and the disease ceased in the beginning of January. Seghill is 6½ miles distant. At Killingworth and Backworth Collieries, about the same distance, it appeared; in the former in the latter end of January, and in the latter in the beginning of February, breaking out with such violence as to produce 70 cases up to the 7th, and 8 deaths.

At Alnwick it was reported to have appeared early in December, but has not there prevailed. At Durham, Morpeth, Stockton-on-Tees, and Doncaster, single cases have occurred, and at Pittington, a short distance from Durham, in the direction of Hetton. In the extensive parish of Houghton-le-Spring the cholera has prevailed at the villages of Houghton, Hetton, Rainton, Penshaw, and Newbottle. The population of the whole parish is about 2,000; of these the township of Hetton contains 999. The first cases occurred at the village of Houghton. This place occupies an elevated situation, and is inhabited by people in comfortable circumstances, but it never prevailed to much extent. At the same time, namely, in the beginning of December, some cases also occurred at Newbottle, also in a high and bleak situation; in the latter end of the month, Penshaw is added to the reports; and, in the beginning of January, Hetton. This place, distinguished by the significant name of Hetton in the Hole, has furnished nearly the whole of the cases reported from the parish. From the middle to the end of January it prevailed here with very great severity, declining in the beginning of February. The numbers again increased towards the middle of the month.

In Middle Rainton, a village close by Houghton and Hetton, the cholera did not prevail extensively until the beginning of February.

HADDINGTON.—This place, selected by the cholera for its first eruption in Scotland, is situated on the bank of a small river, the Tyne, which empties itself into the German Ocean: near Dunbar the North Road passes through it: the distance from Edinburgh 17 miles, and rather more than 100 from Newcastle. The first case occurred here on the 17th of December. A man of dissipated habits, who had been wandering about in a state of intoxication, and almost naked, the previous night, was attacked on the 17th, and died on the 20th December. No other person was seized until Christmas Day, when two other cases occurred, one a girl of seven years of age, the other a woman of dissipated habits. On the 27th three others were seized, and now it prevailed pretty extensively for a fortnight: up to January 9th, 34 cases and 15 deaths had occurred: it declined after this; but 11 new cases and 8 deaths taking place in the succeeding fortnight, up to January 25th. On the 29th of this month it again increased in severity, producing 56 cases and 30 deaths in the fortnight preceding the 7th February.

The earliest cases occurred in a filthy close locality on the river-side, and the debauched, the half-starved, and old persons, as in most other places, constituted the major part of its victims; but, during the first severe irruption several respectable persons died, amongst whom may be particularized the Procurator Fischal, and a well-known school-master. It has been remarked, that this first irruption was confined to the eastern part of the town, to a space perhaps not more than one hundred yards square; but in the second attack that place was entirely forsaken, the disease appearing again in a circumscribed situation, of like extent, in the centre of the town, and amongst those who perished in this attack are noticed a number of highly respectable and opulent individuals. It is also here remarked, that its seizures generally took place in the night, but very few young persons being affected, all those seized recovered with one or two exceptions.

The population of Haddington is 5255, and the severity of the disease has been trifling if compared with many other places in this neighbourhood.

The next place affected was Atherstone-ford about two miles from Haddington. Here two cases occurred on the 8th January; but, after this, it does not appear to have prevailed here.

TRANENT.—This village lies on the North road, about seven miles West of Haddington. It contains about 1700 inhabitants; with the exception of some shopkeepers and other tradesmen, they are chiefly composed of colliers and other labouring people. The first appearance of the cholera here is not very accurately noticed: rumours of its breaking out were first raised on the 15th of January, from the occurrence of several fatal cases together, but afterwards it was recollected that three other cases had previously occurred, which, at the time, were passed by without notice. One of these, an old woman of 70, died, after a few hours' illness, on the 12th; but, as she had been complaining for several years, no particular notice was taken of the disease at the time.

On the 14th a boy, aged 12, of the name of Reid, who had gone to his work as usual in the coal-pit, was there taken ill; he fainted twice before reaching home—his strength became prostrated—the symptoms of cholera followed, and he died on the morning of the 15th. His sister, aged 25, was also at work below ground on the 14th, during her usual hours, and sat up till about 12 o'clock at night preparing a dress, when she was seized with the complaint, and died in the afternoon of the 15th.

On the 15th, a man of the name of Mustard was taken ill, and died the following day. This man was the son of the old woman whose case is noticed above. On the 16th, the father of the children who died on the 15th was taken ill, and a beggar, at the time residing in a low lodging-house in Tranent. The disease rapidly increased, having seized 49 persons, and proved fatal to 20, up to the 23d January; the reports continue to furnish an increasing number of cases up to the 12th of February; the first week after the 23d January having of new cases and deaths, 42, 17; the 2d, 57, 21; the 3d, 64, 25; and the 4th, 91, 12. In the succeeding week, namely, to the 19th February only, the disease declined to 18 new cases and 8 deaths.

We have seen the cholera spreading in the North of England by successive irruptions, and such has been the case in Scotland; for a month had it prevailed at Haddington before it affected the surrounding country, with the exception of the cases noticed at Atherstone-ford; and simultaneous with its appearance at Tranent, Musselburgh was affected, as well as Preston Pans, North Berwick, Biel, near Dunbar, Southfield, and Daddeston. Near Edinburgh, reports of its appearance also came from Dalkeith, Preston Hollm, and Howgate, all on the banks of the North and South Esk, and Hawick, in Teviot Dale. In the course of the next eight or ten days, we find scattered cases from various parts of the surrounding country, of which West Barns, Stenton, and Willingham may be noticed; and, on the 26th, a case at Leith, with 6 on this day and the next in Edinburgh.

On reference to the map, it will be seen that this locality, from 10 to 15 miles in width, occupies the Southern shore of the Frith of Forth, from Edinburgh to the neighbourhood of Dunbar, a distance of 28 miles; a track occupied by fishermen and by colliers, like those in which the cholera has prevailed in the North of England, and, with the exception of the rising ground in the neighbourhood of Edinburgh, towards Dalkeith, low and flat. For this irruption, various concomitant circumstances have been considered the cause. Some dwell upon the revelries of old Hansel Monday, the 16th January, being the first Monday of the year, old style, when the population of Edinburgh poured into East Lothian to visit their friends; others notice an unusual influx of beggars into Tranent during the month of January, driven there by the regulations formed at the different burgh towns, for the expulsion of vagrants; and others, again, remark the occurrence of the disease chiefly among the colliers at Musselburgh, who had struck for wages. Whatever may have been the cause, this disease has raged in Musselburgh, Tranent, and Preston Pans, with far greater severity than hitherto in any other place in the United Kingdom.

From Musselburgh, the first case is reported on the 18th January, followed by 9 on the 19th, 17 on the 20th: up to the 23d, 81 cases had appeared—29 had died. It continued raging with equal severity until the 9th of February. Up to this date, 386 cases had occurred, and 170 deaths; in the next week, the number of cases is 1-3d below the previous average, and, by the end of the month, it had almost entirely ceased.

Typhus fever prevailed amongst the families of the poor before and during the prevalence of this epidemic. The reports give but a very unfair account of the diseases of the season, the greatest care being taken to exclude all but those cases which presented the severer symptoms of cholera. At least an equal number of bowel-complaints, severe enough to require medical treatment, were unreported.

Preston Pans, lying on the coast, between Musselburgh and Tranent, two miles distant from the former, was affected on the 20th January. The first case was that of Jas. Renton, at Preston Grange Colliery: he became unwell about 9 o'clock, p.m. but continued working till about 12; he died the next day. The 2d case was his child, aged about 2—died in 12 hours. The third was the wife of a miner. Of the 6 cases seized on the 22d, one was a carpenter, who made Renton's coffin; another a woman, seized while loooking, from her stairhead, at the assemblage of people at the funeral; two were children of widow Bolton, who attended Renton's family when in distress; the other two were altogether unconnected. Beggars, colliers, and dissipated persons form the majority of the fatal cases. The prevalence of the disease was here progressive, increasing up to the end of the first week in February, and then again declining.

We have noticed the existence of the cholera in Haddington for a month before these places were affected; but a reference to our table will shew, that the agent producing it must have operated simultaneously over this track of country; for it appeared, arrived at its height, and declined at Tranent, Musselburgh, and Preston Pans; the second irruption at Haddington arriving at its height at the same time, and then declining with them.

In the latter part of the time a case appeared at Wallingford, a village half-way between Musselburgh and Tranent.

In the neighbourhood of Haddington, one case occurred at Gladsmuir, 7th February.

At West Barns, two miles from Dunbar, it appeared first on the 25th of January. Five fatal cases had occurred previous to the 10th February; on that day 3 took place; and, up to the 23d, 7, of which 4 were fatal.

It appeared at North Berwick about the same time as at Preston Pans, continued to prevail during the first week in February, up to which time 15 cases and 5 deaths had occurred. After this time it ceased, but a single case happening on the 10th.

Portobello, midway between Edinburgh and Musselburgh, was not affected till the 18th February. The reports from this place are furnished very irregulary. Twenty cases had been fatal up to the 23d of the month.

Dalkeith. Single cases occurred at this place in the latter end of January and through February, but very few persons have been affected.

Edinbungh. At this place, diarrhœa had been prevalent through the Autumn, and typhus fever. We also hear rumours of fatal cases of cholera appearing at times. On January the 27th, the first acknowledged cases of this epidemic occurred. Three perpersons were seized on that day, and the same number on the 28th, half of which proved fatal. Of these early cases, some had not been in the way of receiving infection, others had been at Musselburgh, but it is not said whether they had been there exposed. They were of middle age, or old persons of dissipated habits, inhabiting the confined and dirty closes of the old town. From this time to the 6th January, no other case occurred; up to the 15th, but 6 other persons were seized; on the 16th and 17th of February, 7; and, between this and the 22d, but a single case appeared: in the month of March, the disease again ceased.

The history of the irruption of this disease in the neighbourhoods of Glasgow and London, we must defer to another opportunity.

### Propagation by Contagion.

After all that has been said on the contagious propagation of the cholera; after the positive and arrogant manner in which this opinion has been maintained, we cannot help feeling an intense interest to examine how far it is confirmed or contradicted by the facts presented in the origin and progress of the disease in this country.

First, with regard to its origin; what proof is there of its importation from any foreign source of infection? It is scarcely worth while to notice the absurd reports that were circulated from Sunderland of the infection being brought from Hamburgh, of the first breaking out of the disease among the crews of a vessel from that place who were allowed to ramble about all night on the shore, and its subsequent communication to the inhabitants of the town. These rumours have been sufficiently exposed in the letter from Dr. Brown, published in our last number, and in another from Dr. Ogden in our present.

Whether the first origin of the epidemic be traced back to the month of August, or dated from the seizure of the elder Sproat, no communication of any of the persons affected with any one arriving from foreign ports where the disease prevailed has yet been made out; and with regard to infection having been brought in the clothes-chests of seamen, we may add to the judicious remarks of Dr. Ogden, the observation, that it is not shewn that any person affected had been in any way exposed either by contact or mere approach to these imagined vehicles of contagion, or that inanimate objects, the dead body of cholera patients excepted, were capable of receiving and transmitting the supposed contagion.

Against these idle tales all the facts which have been observed, prove its indigenous origin in Sunderland, viz:—The general prevalence of complaints of the stomach and bowels, diarrhœa especially, and common cholera, not only at Sunderland, but also in various parts of the kingdom:—The earliest appearance of this complaint in particular individuals, previously labouring under disordered functions of the alimentary canal from intemperance, want of food and clothing, or peculiarity of constitution, inhabiting confined and crowded situations, or infirmity induced by previous illness;—while debauchery and drunkenness, and exposure to atmospheric vicissitudes, are found to have immediately preceded its attacks far oftener than communication with the sick.

In like manner, the successive irruptions at Haddington, Kirkintilloch, and London, present proofs of a local origin of the cholera, again refusing to extend itself in the time of most frequent intercourse, and first appearing at very distant places in persons who had not been exposed to any source of infection. These are not exceptions or anomalies which have been witnessed in this country alone; Berlin, Moscow, Vienna, and many other places in Europe and India, gave the same illustration of a local origin;—and the comparison of its rapid extension over Europe, in defiance of all attempts to arrest its progress by cutting off all communication between healthy and sick districts, with the slow intercourse that takes place between them; the comparison of the rapid and unrestricted communication which has all along existed in this country, with the slow, irregular, and capricious extension of the disease here, at once refute all the ridiculous notions which were once maintained of the contagious propagation of the cholera. It is much less infectious than the plague or small-pox, said the abettors of this opinion;—and yet they shut their eyes to the fact, that its rapidity of progress infinitely outstripped the course of these diseases; and whilst a few were allowed to be susceptible in some places, such numbers were suddenly attacked that it was utterly impossible that they could have been exposed to infection, unless, as some have maintained, amongst whom we find Sir W. Russell and Sir D. Barry, that it had been wafted by the winds. The absurdity of this theory is so palpable, that nothing but the arrogance and presumption with which it has been enforced, could excuse a further notice of it. By whom was it formed, and what was the process of false reasoning by which it was supported?

It was formed in the closet, by men ignorant of its phenomena, in contradiction to the observations of all who were practically conversant with the disease. The facts and reasoning of these were set at nought, and all who dared to uphold the contrary opinion were stigmatised as "men who willfully abandoned all the ordinary maxims of prudence and were obstinately blind to the dictates of common sense."

The frequent extension of the disease where communication with the sick, or persons conveying infection from them, had no existence, as well as the failure of cordons and quarantines in arresting its progress, and the still more frequent exemption where the most frequent intercourse has existed, shew that there may well be other reasons than those assigned for the out-breaking of cholera in the Mauritius, or the little extent to which it prevailed in the neighbouring isle of Bourbon, for the immunity of Sarepta, Peterhoff, the family of the French Consul at Aleppo, and some other similar instances collected in the course of a many thousand miles, of a disease which every where abounds with anomalies.

Nor are these the only points on which erroneous conclusions have been formed. It has been asserted, that the adoption of contagion was safest in its practical bearings—a theory from which no harm could arise, if erroneous. The believers in contagion acknowledge that few are susceptible, and that these are individuals in whom bad diet, deficient clothing, confinement in ill-ventilated and crowded situations, drunkenness, fatigue, distress, or previous ill health, have weakened the powers of the constitution. All who have witnessed the misery occasioned by obstructions to commerce, will perceive that its ravages would be increased by measures which increase the number of the susceptible in a greater ratio than they prevent infection; and if it ever arise from other causes than contagion, the numbers exposed to the influence of these causes would be increased beyond all calculation.

So far it is evident that this disease has appeared in various parts of the country from other causes than contagion; but it still remains to be examined, whether its propagation in the surrounding neighbourhood has been owing to this cause; whether or not it has ever become, under any circumstances, contagious? The order of succession in which places near to each other have been attacked, has been irregular and capricious, as its manner of breaking out in distant parts of the country. Thus the hamlets of Houghton le Spring, Hetton, and Rainton, within the distance of little more than a mile of each other, were affected; Houghton, in the beginning of December; Hetton, in the beginning of January, and Rainton, in the beginning of February: Durham, but a short distance from them, has had only two cases. The disease remained confined to Sunderland until the beginning of December, then it broke out almost simultaneously in all directions. Seaham, Blyth, Newcastle, Chester-le-Street, Seghill, and Newark were affected. Percy Main colliery lies close by Howden Dock, and Wallsend, on the Tyne between Shields and Newcastle; at the latter place it broke out in the beginning of January, but did not rage severely: at the former, it appeared in the beginning of the next month, and prevailed with great violence. It is needless to multiply examples. Every part of its course abounds with facts shewing the dependence of this disease on other causes than that of human intercourse. It would indeed be difficult to point out any single place which has been affected early in proportion to the frequency of the communication of its inhabitants with those of any other place where it raged; but if we were to found our conclusions on such facts, we should fall into the same error as that which the exclusive contagionists have previously committed. It is upon direct instances of communication between the sick and the healthy, not on probabilities or possibilities that our reasoning must be founded. The general facts are these; a very great number of persons have been exposed to the sick without being affected with the disease, or communicating it to those whom they approached: a great number of persons have been seized who have not been in any way exposed to emanations from the sick, whether directly from their persons, or indirectly from substances which had imbibed them. Some have had the disease after such exposure, and it has raged particularly amongst families, often affecting, and sometimes proving fatal to nearly every individual composing them. Shall we then say, that the disease has been produced by contagion in the latter and not in the former? To prove the existence of this agent something more is necessary than the occasional seizure of persons who have previously been in communication with the sick; we must know that no other cause can have produced the disease in them, before we are authorised to attribute it to the operation of a something invisible, intangible, and not by any means to be rendered apparent to our senses; the evidence of this cannot be rendered perfect in our present state of ignorance of the general causes of the cholera; and fortunately it is not a question of great importance. Admit the possibility of its being occasionally as contagious as typhus, merely for the sake of precaution, and there is no fear of its spreading from this cause farther than that disease, even if no restriction on internal intercourse were imposed. To remove all fear from the minds of the attendants of the sick ; to do away with all restrictions which operate injuriously on trade; yet to expose no one unnecessarily; to supply the poor with wholesome food and clothing; and to avoid excess of all kinds, especially drunkenness; are the means which both reason and experience demonstrate to be most effectual to arrest the progress of this disease.

### Symptoms.

The cholera in England has maintained a striking resemblance to the oft repeated descriptions of its symptoms in other countries. Thus, the severe vomiting and purging of peculiar characteristic secretions ; the nausea, internal burning at the epigastrium, intolerable weight, anguish, and oppression ; the paroxysms of severe pain commencing at the stomach, and rapidly extending over the whole alimentary canal ; the ardent thirst ; the cramps ; the deadly prostration, anxiety, and dejection ; the conscious feeling of the hand of death ; the failing of the circulation and animal heat ; the peculiar cold sweat ; shrinking of the skin and subjacent tissues ; sharpening of the features ; contraction of the fingers, and prominence of the tendons ; the hollow, sunken eye ; the leaden aspect of the surface, particularly visible in the hands, feet, nails, lips, and the circles around the mouth and eyes ; the black, thick blood, often not to be obtained ; the difficult and slow respiration ; cold breath and tongue ; the whispering voice, and if death comes not in this shape, the fever rapidly coming on, often takes the last stage of typhus. The sudden invasion, speedy death, or, as rapid recovery, have all been witnessed in England, as well as in the North of Russia, and on the banks of the Ganges.*

But this alarming catalogue drawn up from the whole presents no current picture of any individual case. These different symptoms in different persons, vary infinitely in the degree of their severity, from the common diarrhœa, with little pains and no cramps, and no greater affection of the circulation and temperature of the surface than occurs from the operation of an ordinary purgative, up to the sudden attack of a prostration so alarming, that the sick man becomes scarcely sensible of pain when the secretions are retained and the heart's action sinks at once.

To illustrate these different forms we have made the following selection of cases.

The following bears a greater resemblance to common cholera than to the severer cases of the present epidemic.

Mr. A. Hopton, Deputy Governor of the Goal at Durham, age 58, of rather free habits ; general health not very good. On the 14th of January, he felt oppression at the stomach, sense of heat, and uneasiness, with occasional nausea. At two o'clock on the morning of the following day, he was seized with violent purging, attended with most distressing cramps in the limbs : the evacuations were almost incessant throughout the day ; thin and watery, of a pale white colour, almost without smell ; at eight in the evening, there was great alteration in his appearance ; countenance blanched and anxious ; features sharp ; skin rather cold and covered with a clammy sweat ; pulse slow and weak ; breathing oppressed ; complains of most distressing thirst, some softning of cuticle of fingers ; the cramps are not constant, but recur at intervals ; most violent in the hands and feet ; occasionally extending to the thigh ; inclined to sleep in the intervals.

*Treatment.* Coffee, mustard, brandy, opium, cayenne pepper, camphor, carb. ammonia, and oil of peppermint. The symptoms continued somewhat mitigated on this day and the following.

The premonitory symptoms have been, in some cases, of long duration ; in others absent. They are uneasiness ; a sense of heat and disorder in the stomach and bowels ; frequently diarrhœa, which at first presents no peculiar character, so that no man could tell whether an attack of cholera was impending or not ; this has prevailed, in some cases, for a week or more before the attack, in others, it has only preceded the other symptoms for a few hours, or a shorter period.

The elder Sproat, aged 69, the first case of acknowledged cholera in Sunderland, had been labouring under diarrhœa a week or ten days before his seizure. On Wednesday, Oct.

---

* We think that certain documents in this number, will shew that a very important modification of the disease has taken place since its first irruption in India—a modification that inculcates considerable difference in treatment.—Ed.

19th, he had been taken worse: on Thursday and Friday, he had vomiting and purging of fæculent matter, but no symptom of collapse. On Saturday he was greatly better, took a mutton-chop to his dinner, and went out to his keel in the afternoon. In about 20 minutes he returned, and was taken very ill, with severe shivering, giddiness, cramp at the stomach, violent vomiting and purging. On Sunday morning he was sinking; pulse imperceptible; extremities cold; skin dry; eyes sunk; lips blue; features shrunk; whispering voice; violent vomiting and purging; cramp of the calves of the legs, and complete prostration. In the afternoon his skin became warmer, but the other symptoms continued. On the 24th he was quite collapsed, with aggravation of all the symptoms except the vomiting, which had entirely ceased; stools passed involuntarily. On the following morning he was less collapsed; countenance more natural; blueness of the lips had disappeared; the vomiting had ceased; but the purging still continued less violent, and nearly imperceptible; extremities cold; spasms of the legs continued. Towards evening, the purging and vomiting had entirely ceased; he became sleepy; the other symptoms continuing. On the morning of the 26th he was much weaker; pulse scarcely perceptible; countenance quite shrunk; eyes sunk; lips blue, as well as the lower extremities; the nails were livid. He was comatose, and died at 12 at noon.

2. *Premonitory Symptoms, slight, and of very short Duration.*

Susanna Clark, aged 18. December 5th, about 5 in the evening, she complained of uneasiness and distention of the stomach and bowels: her countenance became pallid, and expressive of much anxiety and distress. She was attacked with vomiting and purging of bilious fluids, and with cramps. She continued in this state until 8 in the evening, when bleeding was unsuccessfully attempted. She took brandy, and a mixture containing laudanum, capsicum, and ammonia. The vomiting ceased, she became much better in the night, and, on the morning of the 6th, her pulse was full and her body warm, complaining of little except a pain in the head: but, about midnight, the cramps, vomiting, and purging returned; she became cold, and apparently almost lifeless, though still sensible. Her pulse was gone; her eyes deeply sunk; she remained in the same state through the day, until 6 at night, when she became comatose, and died at 8.

3. *Premonitory Diarrhœa, of short Duration.*

Isabella Carr, aged 6. Went to bed in good health. During the night she had some diarrhœa. At 8 o'clock on the morning of the 11th Dec. she was attacked with severe vomiting of a watery fluid and purging of liquid matters, tinged with bile of a faint fæcal odour. At noon, she was without pulse, cold, her hands blue, eyes much shrunk in their orbits, the vomiting and purging still going on. She died at 3 in the afternoon.

In the last case, purging continued nearly up to the time of death; in others, which constitute the majority of the fatal cases in this country and in others, the vomiting and purging soon give way to alarming prostration.

Thomas Wilson, a keelman, æt. 51, was attacked, on the morning of 31st October, about four o'clock, with vomiting and copious purging of a fluid resembling rice-water, accompanied by severe abdominal pain and spasms of the extremities. At 6, carb. ammon. laudanum and warm bath. At 7, vomiting ceased; one dejection, resembling rice water, of a peculiar sickening and highly offensive smell, something like putrid animal matter. Complained of intense pain in the epigastrium and abdomen—cramps of the extremities—extreme restlessness, moaning and sighing. Pulse imperceptible at the wrist—surface of the body cold as death—lips blue—eyes dim and sunk in the head—speaks in a whisper, intellect clear—tongue moist and cold—respiration slow—urine suppressed: the spasms ceased about 9—no vomiting or purging. There appeared a total loss of power of the nervous and circulating systems; at 12, the appearance of a living corpse—eyes deeply sunk in their sockets—hands and fingers remarkably shrivelled, very much reduced in size, and of a light blue tinge. He gradually got worse, and expired at three o'clock in the afternoon.

E. Turnbull, a strong healthy woman, was taken ill 1st November, about one o'clock. Between four and five the following symptoms were present: violent vomiting and purging of a watery fluid, in appearance like oatmeal gruel; excruciating spasms in the muscles of the legs and arms; pulse only perceptible at one wrist; voice puerile; tongue cool; extremities cold, and of a livid hue; fingers and toes much shrunk; intellects perfectly clear; complaining of pain at the region of the stomach, and calling for cold water. A vein was freely opened in each arm, from which flowed only a few drops of blood, like treacle: external and internal stimulants, with opium, were freely used, without effect. About 10, a.m. the cramps had nearly ceased: the skin became universally cold as marble, and, at the epigastre, of a deep purple hue. From this time till 2, p.m. the period of her death, the only symptoms of life were a gentle heaving of the chest, a rational answer being given to any question proposed to her.

The following case presents a little variety in the jactitation

Maria Mills, æt. 42. Attacked at half-past 10, 7th Dec. with vomiting, purging, and cramps at the extremities. At half-past 4, p.m. the pulse was imperceptible—cold, clammy skin—spasms of legs and toes—breath laborious—thirst excessive—integuments of fingers and toes corrugated and softened—no more evacuations. Half-past 8. Increased labour in breathing—tongue colder—head and arms livid—nails dark—perfectly sensible. Complained of no pain, but shrunk from pressure at the epigastrium; jactitation, frequently changing her position with considerable quickness and force; every moment seemed the impulse of some convulsive efforts; loss of deglutition; death, 12¾ hours from commencement.

William Bell, æt. 67, was taken ill at 8 o'clock at night, December 2d, with cramps in the legs, vomiting, purging, and coldness of the whole body. No pulse could be felt at 10; oz. ij. of blood only could be obtained; he passed a restless night; no pulse; the body cold and damp; eyes sunk; cuticle of hands and feet corrugated and bluish; tongue warm; lies without stirring, and when asked where he feels pain, *lays his hand on the epigastrium and complains of a great weight* on the same region. These were the symptoms at 11, a. m. 3d. He died at 4 in the afternoon.

Susan Roach, æt. 40. She was taken ill at 8 in the morning, 8th Dec. with cramps, vomiting and purging, great uneasiness at epigastrium. At 9 in the evening, when first seen, her extremities were cold and livid, but the surface of the trunk was not much below the natural temperature. Tongue and breath cold; pulse perceptible; four ounces of blood were with difficulty taken away. She was better on the 9th and 10th, and convalescent on the 11th. The treatment was brandy and other stimulants, dry heat and friction to the surface.

### *Severe Case in a Child.*

Daltel, æt. 2½, had slight diarrhœa for about a week; had been playing in the lane in the day. About 7 o'clock in the evening, Dec. 1st, he was seized with severe vomiting and purging; the surface of the whole body speedily became cold; was very restless during the night. At 11 o'clock in the morning of the 2d he was lying motionless on the lap of his aunt, his skin cold and damp, *no pulse, no vomiting or purging;* his eyes sunk in their orbits, and surrounded with a dark and broad circle; his lips, and the parts of his face around the mouth, and the hands, of dirty purplish blue colour. At 7 in the evening he died.

### *Absence of Premonitory Symptoms.*

Mrs. Fairly, æt. 61, chief nurse of the cholera hospital at Sunderland. She had eaten her breakfast with her usual appetite, and may be said to have continued in perfect health up to the moment of attack. At 11, a. m. Dec. 2d, she was attacked with purging and some vomiting; the fluids ejected soon exhibited the characteristic appearance; she had pains in the epigastrium and general uneasiness; the vomiting and purging had continued for an hour when the countenance indicated great depression, the voice was sinking, pulse scarcely perceptible, skin cold and damp, with severe spasms of the muscles of the legs and thighs; she had taken some pills of calomel, opium and capsicum on the commencement of the attack, and now she was bled to about 20 ounces, *after which* the pulse almost completely disappeared at the wrist, the vomiting and purging ceased, but the symptoms of collapse increased; the pulse quite gone, skin cold and clammy, the eyes sunk, and surrounded by a dark ring, great thirst, moaning, restlessness, complains of severe pain in the epigastrium, and utter dejection of spirits. At six in the evening the pulse became slightly perceptible; the great pain and oppression at the stomach continue; she fell asleep at seven, but awoke soon afterwards in a state of great suffering; was restless, scarce any pulse, at times much convulsed; was sensible though she does not speak; deep blueness of the hands and feet; the cuticle of sodden appearance. Died about a quarter after eight, after nine hours' illness.

*Long Duration of the Stage of Collapse.*

Jane Todd, æt. 54 : At 8 o'clock, p. m. Dec. 4th, she was suddenly taken ill with vomiting, purging, and cramps. At 11, a. m. 5th, the pulse had ceased, the general coldness of surface was present, the sunken eyes surrounded with a dark ring, and sharp features; lies quiet, unwilling to be disturbed; the vomiting, purging, and cramps have ceased. She continued in this stake, making no complaints, sleeping a little at times, until the morning of the 7th, when she died; the only alteration in the last 24 hours being the return of some warmth to the surface, and a slight fluttering in the pulse.

*Slight vomiting and purging, resembling the cold stage of a remittent more than Cholera.*

Susan Hanson, æt. 19 : At 2, p.m Nov. 29th, she was attacked with shivering and coldness of surface, cramps in the left arm and leg; some, but *not severe*, vomiting and purging, and pain in the head. At 8 there was a general coldness of the surface, a feeble pulse and cramps; at 12, pulse 98; tongue moist, clean, and warm; great thirst; cramps in the arms; hurried respiration; great pain and weight at the precordia; nausea, but no vomiting; pain in the head and vertigo: bleeding relieved the breathing, external warmth was applied. In the evening she was much better, had no cramps, vomiting, or purging; on the following day only a little pain at the stomach remained.

*Evacuations trifling; no Sweat; slow Course; Fever little developed.*

E. Scott, æt. 50: During the night she had some vomiting and purging, but not severe; evacuations resembled rice-water or thin gruel. At 11, p. m. no pulse at the wrist, tongue and breath cold, body cold and dry. Continued in the same state until the afternoon of the following day, when very trifling reaction occurred, and the pulse became perceptible. 3d day. The skin of the trunk warm and dry, hands and feet cold, tongue dry and brown, pulse 70, easily compressed; bowels relaxed, dejections contain bile. 4th day. Pulse very feeble and slow, skin dry, tongue dry and brown, speaks occasionally, but unless addressed she lies in a comatose state, refusing everything. 5th day. Remained in the same condition; at times in a state of coma, but occasionally very restless. Died in the evening.

*Long Duration of Purging—Slow Course of Fever.*

Isabella Elliot, æt. 5 : Taken ill on the morning of the 24th with spasms, vomiting, and purging; the stage of collapse came on rapidly at 10 o'clock; her extremities were very cold, her countenance and fingers blue; eyes deeply sunk in their orbits; still vomiting and purging; fluids like rice-water; in the evening the breathing was difficult, the skin became warm, the countenance improved, no cramps, but pain at the stomach. Nov. 25th, noon, except purging and eye remaining sunk, all other symptoms favourable. In the evening reaction was fully established, the surface became hot, pulse rapid, respiration free, eyes lively, restlessness; the following morning much improved and pulse still quick; some bile is observed in the dejections; (had rhub. and mag. and turpentine injection the previous night.) Nov. 27th. Much purging, conjunctiva much injected, restless, pulse quick; in the evening breathing difficult, skin hot, spasms of the abdominal muscles, conjunctiva becoming dim and opaque, lips dry, gums covered with black sordes. Died at 4 in the morning of the 28th.

The father of this child affords a case of long duration of the succeeding fever. Thomas Elliot, æt. 34 : At 2 o'clock on Monday morning he had severe symptoms of collapse, with cramps and purging at intervals. After inhaling two large bladders of oxygen gas, the pulse returned to the wrist: half an ounce of liq. ammoniæ was given and repeated in half an hour. At 10, a.m. the cramps, vomiting, and purging had ceased; the pulse was perceptible, but the face still collapsed, and the breathing difficult. At noon, the pulse increased in rapidity, and some symptoms of fever appeared. Stimulants were omitted. The next day the fever was increased, with pain of head, and great restlessness. 30th Nov. It had assumed a bad form of typhus; no urine has been passed: during his illness; the bowels are soft and free from pain; the dejections contain bile. He gradually became worse, the eyes much injected, delirium continuing, and on the night of the 2d December he sank into a state of coma, from which he was with difficulty roused. Died on the night of the 4th, having passed no urine since the 28th November.

*Recovery from Consecutive Fever.*

Isabella Cowan, aged 10, was taken ill Nov. 25th, with vomiting and purging, having previously had slight diarrhœa. On the following morning her pulse could scarcely be felt; she had no cramps; her tongue, breath, and surface of the body generally, were cold; her fingers and lips blue. Treatment directed by Dr. Ogden—mustard poultice over the stomach, two grains of capsicum, one of calomel, and ¼ of a grain of opium. Every hour warm brandy and water; dry heat to the body, and diligent frictions with hot flannels. In the evening the purging of fluids like rice-water continued, and in the dejections were many ascarides; her skin was warm, her colour and circulation improved. Nov. 27th, the pulse was full, the heat of body natural. An enema containing some ol. terebinthinæ was given. The following day she gradually sunk into a state of fever resembling typhus, attended with delirium. She was slowly recovering from this through the use of mild aperients, cold applications to the head, and blisters to the nape of the neck, when a relapse was occasioned by her mother giving her some bad pastry. On the 8th December she was quite well, excepting some debility.

We may add a case in which the evacuations were very profuse, and another in which the cramps were severe.

Betty Short, æt. 45: Had suffered in the afternoon, Nov. 7th, from distention of the bowels and general uneasiness, for which she had taken calomel, extract. colocynth. and salts, with infusion of senna. At 10 at night she was sitting by the fire, drawn up in severe cramp, vomiting profusely a mucous watery-looking fluid, tinged with the ingesta; copious dejections (in fact a pailful) of bilious-coloured liquid. Extremities cold and livid; pulse 100, scarcely perceptible, and extremely small; tongue cold; great restlessness and anxiety. The next day the cramps disappeared, the failure of the circulation and prostration increased, the purging diminished; brandy was constantly rejected. On the 9th the pulse became perceptible; in the morning the vomiting and purging ceased, but the prostration increased: at night the pulse again became imperceptible. She died at half-past 12, p. m.

2. *Case in which the Cramps were Severe.*

Haddington.—Samuel Parson, aged 42, had been attacked at half-past 3, a.m. 31st December, with vomiting and purging, accompanied with pain of abdomen and much thirst; to allay which he had taken frequent draughts of cold water, vomiting them as soon as taken. Said to have had three alvine evacuations. The countenance is of a livid ashen hue; features shrunk; eyes deeply sunk, surrounded with a livid areola, half closed and turned upwards; breathing difficult; much oppression at the chest; headache; tongue white; severe thirst; urgent craving for cold water; hands covered with cold sweat; severe cramps in the legs, and spasms in the arms and fingers; pulse not to be felt at the wrist, and but indistinctly far up the humeral artery: 25 minutes after 7 the vomiting had ceased, but he continued to retch frequently. Coldness of surface increasing; cramps continue severe; prostration rapidly increasing; at 9 cold sweat all over the body; no motion or vomiting; unable to swallow at ½ past 9; died at 10 minutes past 10 o'clock.

The treatment was bleeding to the extent of six ounces; sinapisms with oil of turpentine to the abdomen and feet; friction of the legs with oil of turpentine; a drachm of laudanum and æther in a draught; a wine-glass of brandy and hot water to be repeated every half-hour.

In these different varieties of the severe forms of cholera we have very copious evacuations without the peculiar collapse, and the latter sometimes without the precession of copious evacuations; in other cases it has succeeded these after various intervals of time, and has also occasionally preceded them. We have seen some recover from this alarming state without going through any consecutive fever; others pass from the one into the other, proving fatal to most, but not to all.

In some the vomiting is almost absent, and in others is very profuse and frequent: the same is true of the purging. The cramps at times form the most distressing symptom; at other times they are absent. The pulse has been found greatly retarded as well as quickened; the respiration occasionally slow and very difficult, but the strongest marked failure of the circulation has taken place whilst respiration remained free: and all these different forms are met with in children of tender years, as well as in the aged; in males as well as females; notwithstanding some varieties occur more frequently in particular constitutions than in others.

In selecting for illustration of its symptoms, the severest cases of the present epidemic, we have followed the beaten track; one which leads to very erroneous impressions of the disease in general. Cases of this severity form but a very small proportion of the whole; the greater number have a closer resemblance to the ordinary forms of common cholera; and still more prevalent have been disorders of the bowels, scarcely if at all differing from ordinary diarrhœa—in every possible grade of intensity, from the slight fæculent relaxation which has not kept the person labouring under it from his usual occupation, up to the copious and frequent purging of reddish-colored watery fluids, attended with much prostration. It has also been observed, that the dejections have frequently presented a greater resemblance to those of cholera than diarrhœa, that is, more fluid, whiter, and less fæculent than they usually are in the latter complaint.

All these different forms of disorder are found to run so much into each other, that no proper line of distinction exists in nature. We have observed in the details of the preceding cases the frequency of diarrhœa as a precursor of cholera, which, Dr. M'Cann observes, "in a great majority of cases, presented for a time no peculiar character; so that no man could tell, when called early to a patient labouring under it, whether an attack of cholera was impending or not." Nor have these things been peculiar to the epidemic in England; the reports of Drs. Russell and Barry from Russia, and various others from different parts of Europe, all observe the same.

Although it is very seldom that the comparative prevalence of these different forms of disease are reported, in the observations of Dr. Kumes on the cholera, at the Mauritius, which we have already had occasion to notice, as the most complete of any which have been published, we have the following table:—

| | Nov. | December. | January. | Total. |
|---|---|---|---|---|
| Cholera Lethalis | 2 | 12 | 1 | 15 |
| ——— Mitior | 2 | 28 | 11 | 42 |
| ——— Diarrhœa | 2 | 7 | 6 | 25 |
| ——— Vomitus | 5 | 17 | 1 | 23 |
| ——— Signa Anomala | | 30 | 3 | 33 |
| | | | | 138 |

These cases occurred in the 56th Regt. the number of which remaining at Head Quarters were, in Nov., 617—Dec., 593—Jan., 617. Dr. Kumes observes, we may perhaps safely assume, that they all depended on the same cause, modified in its operation by peculiarities of constitution, habits of life, and circumstances of exposure.

In the reports from Sunderland, for a short time, cases of diarrhœa were returned; and although some of cholera were included in them, there is sufficient reason to believe the diarrhœa was actually more frequent than both the milder and severer forms of cholera. By the orders of the Board of Health, no returns of any but those cases which really assumed the forms of cholera have been made; hence we are deprived of much very important information: but that diarrhœa did continue to prevail along with cholera in other places, is quite certain. At Musselburgh, we are informed, that the greatest care was taken to admit none on the report, unless labouring under vomiting and diarrhœa, with spasms, or reduced to a state of collapse; and that while 416 cases only, had been reported as cholera, it was computed that nearly 1000 had been under medical treatment.*

### POST-MORTEM INSPECTIONS.

Dissection of the subjects dead of this disease, has not been frequently performed: the same prejudices of the ignorant multitude have forbidden it here, as in all other countries. Yet so far as opportunities have been afforded, and these have been few indeed, the same general appearances, and similar varieties, in different cases, have been observed in this country.

The entire absence of fæcal matter in the contents of the intestines; the presence, in greater or less quantity, of matter such as the peculiar evacuations; the serum-like fluid, more or less abounding with floculi of coagulated albumen, which are occasionally

* Since that period diarrhœa has been pronounced by the Board as the *first*—and we may add, as very often the last and only stage of cholera.

found in such quantity, as to line the mucous membrane with a tenacious substance, like paste; an odour of putrifying mucus, somewhat like that of offensive lochial discharges, occasionally present; the serous fluid being frequently more or less tinged with blood, and that of the stomach often mixed with the latest ingesta; the upper part of the duodenum often slightly tinged with bile; frequently contractions of different portions of the colon; occasionally introsusceptions; the gall-badder distended with unhealthy bile; its ducts sometimes strictured, and the urinary bladder empty and remarkably contracted.

The mucous membrane of the alimentary canal generally somewhat softened; sometimes of an unnatural paleness throughout, but oftener having various portions tinted of different hues, from the pale rose, to dark brick-dust and slate colours, as venous or arterial injection predominates; patches of ecchymosis and arborisations of the larger branches are frequent; but the most common appearances have been a red or purplish speckling of the membrane, generally over the whole, or more apparent in some parts than others; or an injection confined to the prominent parts of the rugæ of the stomach and valvulæ conniventes of the intestines. Sometimes these different appearances are scattered throughout the whôle extent of the mucous membrane; at other times the stomach alone is coloured, and the intestines pale, or the stomach pale, and different portions of the intestines darkly injected; however, these appearances are most frequent about the smaller extremity of the stomach and lower portion of the ileum. The venous trunks of the stomach and intestines are generally found greatly engorged; such also frequently takes place with the liver and spleen, and almost in every case in the venæ cavæ, and auricles of the heart. The blood remaining fluid, of an exceeding dark and peculiar consistence and adhesiveness, likened by some to treacle poured over the cavities of the heart. The lungs frequently exhibit a remarkable shrinking, like that of the skin and subcutaneous tissues (which led in India to the body being opened under water, to ascertain if air existed in the cavity of the pleura), then blood of the same dark hue; frequently they are engorged so as to resemble hepatization somewhat, but still crepitous and lighter than water. The obstruction of the bronchiæ by peculiar secretions has not here been noticed amongst the cases from the North of England: the brain of two only has been examined; in one the vessels were very turgid, and half an ounce of serum was contained in the lateral ventricles; in the other, a very highly injected state of the vessels external to the brain, and in the cortical substance of the upper parts of the hemispheres, and in the cortical substance or upper part of the medulla oblongata was found, with depositions of lymph upon the surface of the brain and cerebellum, and five or six ounces of serum in the base of the skull.

Turgescence of the vessels of the brain and spine has been at all places a very frequent appearance, and serous effusion beneath the arachnoid in the cavity of the cranium and spinal canal, and in the lateral ventricles. It has seldom been observed how often these appearances have existed; but in the report of Dr. Kumes, from the Mauritius, decidedly the best of any which have yet been published, as affording the greatest extent of information on the many important considerations connected with the cholera, in 13 cases, where the brain was examined, serous effusion was found in all but two, one of which appeared natural, the other was mangled by an officious orderly during a momentary absence of the operator, and in one half of the cases where the spine was examined there was effusion under the dura mater of the cord, and the veins generally turgid.

It has been a general complaint, that the state of the organs after death has thrown no light on the nature or causes of the disease, and that they afford no explanation of the severity of the symptoms. It is true that they do not explain these, as the inflammation, tumefaction of the membrane, and fibrinous effusion in croup does the peculiar affection of the voice, and suffocation met with in that malady; but they do throw as much light, as post-mortem examinations in general, on the seat and nature of diseases; and this instance may serve as a useful comment on the present mania for the exclusive cultivation of morbid anatomy: we feel and acknowledge its great advantages, and the precision in this obscure science, which is derived from such researches; but the man who wishes to treat disease successfully, must not consider his only aim and object to be the knowledge of the various morbid changes which take place in the structure of different organs, and of the symptoms by which they are distinguished; his researches must be directed beyond this—to the laws of vital actions; to the manner in which they are disordered, and to the agents capable of restoring them to their healthy state.

We regret exceedingly that any idea of post-mortem examinations being of little utility in investigations into the nature of the disease should have thrown a damp on the interest with which they have been prosecuted, and thus have deprived us of much valuable information. It is, unquestionably, important to ascertain if any connexion exists between the various fatal forms of the cholera, and the different pathological conditions of the organs affected: but on this subject little or nothing has been added to the observations contained in the Bengal Report; namely, that in the bodies of those who died in the earlier stages of the disease, hardly any unhealthy appearance was observed, the intestines, stomach, &c. being paler than usual:—but in the more protracted cases, a greater or less degree of injection of the mucous membrane, with occasional ecchymosis, was the most frequent appearance. It has also been observed, in some cases, where the violence of the spasms proved the most prominent symptom, that the mucous membrane exhibited that appearance, approaching nearest to inflammation. This interesting and important enquiry remains open to those at present engaged in investigating the nature of this disease, and we hope to be enabled to lay before our readers much valuable information on the subject, in our next number.

The relations or differences existing between the common cholera and the present epidemic, is the subject which next claims our attention.

Nothing is more common than to hear different practitioners assert, that this disease resembles none with which they were previously acquainted. We give them full credit for the sincerity of this assertion, and at the same time take the liberty of asking, what was the extent of their previous knowledge of all the different forms and grades of the common cholera. This question will best be answered by a comparison of the symptoms of the two diseases.

The diagnostic was first founded on the rice-water appearance of the evacuations:—those of the common cholera were supposed to be always bilious. This was an egregious error, although committed by Cullen. Later observations have shewn, that evacuations, of the precise character of the former are common in the latter disease. We refer to various cases published in the medical journals since this question became agitated, and we extract two others from the very valuable work of Mr. Thackrah.

"In September, 1825, a debilitated female, attended by Mr. Corsellis, was seized at three a. m. with vomiting and purging. Most distressing cramps speedily ensued; the surface became cold; the countenance sunk; and, to use the phrase of the woman who attended her, 'was all blue as violet." *The stools were colourless*—she could not retain them. She died about 8 in the evening, 17 hours from the commencement of the urgent symptoms. On laying out the body, the women particularly remarked its blue, black, mottled appearance. *One leg remained flexed by spasm, its foot resting on the shin of the other.*"

It is of little importance now to trace the origin of this false fact, but we may remark that the same error and contradiction is of very ancient date. Hippocrates,* Celsus,† Cœlius Aurelian‡ and Galen,§ all derive the word cholera from χολη, yellow bile. Celsus says "intestina torquentur, BILIS supra infraque erumpit, *primum* AQUÆ similis, *deinde ut in ea recens caro lota esse videatur*, interdum alba, nonnunquam nigra vel varia. Ergo eo nomine morbum hunc χολέραν Græci nominarunt."

This "aquæ similis" puzzled Alexander Trallian,|| who in consequence seeks another derivation from χολαδες, intestines, because, he says, bile is not always ejected, but *frequently even a serous and pituitous humor*. These discrepancies Riverius attempts to reconcile by arguing that, although bilious humors are not always ejected, yet they are acrid, corrosive and corrupt, approaching the nature of bile. Areteus remarks, that the dejections are pituitous and sometimes bilious. After this time observation slumbered on the question, until the alarming violence of the present epidemic again raised the agitation.

Another diagnostic has been formed on the frequent precurrence of diarrhœa. The common cholera, says the advocate for this, always makes its attack suddenly—we are again obliged to Mr. Thackrah¶ for the means of refuting this assertion.

---

* Hippocrat., 7 Epid. Sextus 19. † Celsus, B. 4, ch. 11. ‡ Cœlius Aurelian, lib. 3, ch. 9.

§ Galen, 1 Meth., c. 2. || Alexander Trallian, lib. 7, ch. 14. ¶ Thackrah, p. 34.

"In July, 1822, a stout healthy man was affected with laxity for about a week. It was not such, however, as to prevent him pursuing his usual active occupation. After chapel, on Sunday, he ate a dinner of rice, and drank a mug of beer; but half an hour afterwards, about one o'clock, he was seized with copious vomiting and aggravated purging. The dejections were light colored and muddy. In an hour his face was contracted, cold, and livid, and, to use his wife's phrase, 'nipped like death,' and the circle of his mouth was purple. Thinking her husband dying, she sent an urgent summons for medical assistance. Visited between 3 and 4 p.m.; he was in a state of great exhaustion; suffering from severe cramps; his countenance sunk, haggard, and purple; pupils contracted; surface of the body unusually cold, and covered with a clammy sweat; pulse not to be found at the wrists, and in the carotid beating weakly, 155; voice lost; evacuations from the bowels wholly destitute of bile. Mr. Corsellis, then my pupil, remained with the man all next day, assiduously employing the remedies prescribed. For 7 hours there was no improvement, no increase of temperature, no pulse at the wrist, and voice audible only when the inquirer's ear was close to the patient's mouth. After this time, however, there was a slight re-action, and this progressively advanced. Stools were procured of the colour of gingerbread. It was particularly remarked that, for three days from the attack, no urine was voided. This man is alive and healthy at the present time."

"My excellent friend and quondam pupil, Dr. Whytehead of Beverly, has referred me to a case which occurred while he was with me, in the year 1825, and of which I regret that I cannot find the details recorded. A man was attacked with violent symptoms of cholera at 2 o'clock in the morning, and sunk at 10—eight hours consequently from the invasion of the disease. On post-mortem examination, our principal remark was the large quantity of albuminous matter in the small intestines. The stomach was greatly contracted."

Nor ought we to pass by the observation, that although, in the present epidemic, a spasmodic stricture of the gall-ducts generally prevents the intermingling of the bile with the other secretions from the mucous membrane of the alimentary canal, yet such is not always the case. The Indian reports, and all others collected from an extensive series of observations, afford examples, not unfrequent, of the evacuations being more or less coloured with bile, and sometimes even very darkly. We ought not to forget that the disorder of the biliary secretion, whether retained or evacuated, is at least as freqent in this as in the common cholera, the greater or less distention of the gall-bladder with dark *green* unhealthy bile being one of the most invariable of the post-mortem appearances; and profuse evacuations of this fluid are one of the most frequent occurrences in recovery from the present epidemic. The following conclusions appear to us to be correct. 1st. That in both the abovementioned diseases, the secretion of bile is variously affected in different cases; in some, greatly disordered in appearance and quantity, in others not perceptibly: for we have witnessed cases of cholera, of great severity, in which the peculiar sero-flocculent secretions have been present, without being followed, on recovery, by any evacuations marking the presence of bile, unusual in either quantity or colour; and such cases have also been observed during the present epidemic. Secondly, if there is any difference in these respects between these diseases, it lies in the greater frequency of the spasm of the gall-ducts in the one than in the other.

The quantity of the discharges has also been considered an important distinction; the buckets-full of the secretion in common cholera have been pointed out, in opposition to the small quantities evacuated in many fatal instances of the other disease, forgetting, at the same time, that the latter has presented cases, where the evacuations have been as profuse as in any of the former; and that, even here, this symptom is met with in nearly as great variety.

If any one feels dissatisfied with the evidence of the single cases which have been brought forward, we can shortly collect him more. At present it is necessary to economize time and paper.

For the ignorance of the different forms and varieties of common cholera which has hitherto prevailed, the following facts afford a satisfactory explanation. First, this disease is, as compared with others, of unfrequent occurrence; the experience of almost every practitioner not affording him the opportunity of witnessing all its varieties. Secondly, the most plain and palpable phenomena are usually passed by unnoticed, until some question arises to direct the attention to them.

Besides this reasoning, in ignorance of facts, another very considerable source of error has arisen from the practice of selecting, as standards of comparison, the severest forms of the present epidemic, and the milder ones of the cholera misnamed common. Thus, Dr. Barry (Reports of Debates at Westminster Med. Society) is reported to have pointed out, as characters which certainly distinguish the former of these diseases, the remarkable blueness, cold breath and tongue, extraordinary look of the eyes, sunk in their sockets, which appear excavated, excessive corrugation of the skin, tendons of the hands and feet as prominent as if all the intervening substance had been dissected out, which he had observed in cases of the present epidemic, and observes that, if seen before, they would have been described by Sydenham, Celsus, and others. We shall not attempt to explain why they have not been noticed by these accurate observers, or why medullary sarcoma, and many other equally remarkable diseases, escaped their notice; it is sufficient for us to shew that others have described such symptoms, if they have not.*

"In 1829, a young gentleman, after a few days' bowel-complaint, which was not severe enough to keep him from school, was suddenly seized, early in the morning, with great debility, vomiting, and fainting. When I reached the house, his face *was contracted, purple, without warmth,* and expressive of distress. The whole surface was cold; at the wrist no pulse could be found; in the carotids it was feeble. There was then neither vomiting nor purging; but the cramps were particularly severe, and he complained of great oppression at the stomach. About two hours after active treatment, the circulation rallied, and in the afternoon the urgent symptoms had subsided. He remained, however, for several days in a state of great debility, and it was longer before a healthy state of the secretions could be restored."

"A female, about 25 years of age, of a slender make, and dark complexion, who had been previously subject to attacks of pain at the stomach, was seized between one and two o'clock in the morning with sickness and pain in the stomach and bowels, purging, succeeded by vomiting. I saw her about nine o'clock. The countenance was pale, respiration feeble, pulse 110 and weak, the legs cold and occasionally affected with cramp, the sickness, vomiting, purging, and pain unabated. The matter last vomited was copious, consisting of a mucous fluid containing flocculent matter of a greenish tinge; the motion was scanty, of a mucous fluid similar to that yielded from the stomach, but the flocculi it contained were yellowish; no fæcal smell could be perceived. She had made no water since the commencement of these symptoms, when a quantity of limpid urine had been passed. At two o'clock the symptoms continued unabated, the forehead was cold and damp, the eyes sunk and surrounded by a dark circle, the lips livid, the tongue a little furred, moist and cold, the hands pale and shrunk, the abdomen tender to the touch, and feeling as if it contained firm elastic masses. The least raising of the head from the pillow occasioned sickness and vomiting. The matter vomited was similar in appearance to that previously examined, with the absence of the greenish tinge of the flocculi; and a scanty stool, the only one preserved since the morning visit, was not distinguishable in appearance from the vomitings. The cramp had increased in frequency and force, the pulse was 130, and hardly perceptible at the wrist, the voice was tolerably firm. About six ounces of inky blood were with much difficulty obtained from the arm. It coagulated soon, was a little buffed and tolerably firm. After the use of active remedies, a copious vomiting, containing some alimentary matter, succeeded, the pain ceased, the pulse rose to 140, the countenance became coloured, and a calm, accompanied with a few minutes of sleep, succeeded, during which the orifice in the arm poured out a considerable quantity of florid blood. At seven o'clock in the evening I left my patient relieved of all her urgent symptoms. The vomiting occurred twice during the succeeding night, but did not occasion any distress, and she gradually recovered from her state of exhaustion."—*(Thackrah.)*

It is apparent that no symptom or combination of symptoms, have been described in the one, which have not also been found in the other. Has the present cholera been accompanied in its course by diarrhœa and fever? so has that cholera with which we have been always acquainted! The farther we examine into the nature of these diseases, the constitutions susceptible of their influence, and the circumstances which excite their attack, the stronger we find the evidence of their identity in all things, except the extensive range and augmented severity of the present epidemic, from which the correct inference to be drawn is, not that this disease differs in nature, but in the degree of its severity; not that it is produced by different, but by more widely diffused and more violent causes.

* See our extracts from Frank and others.—*Ed.*

### Ratio Signorum.

The peculiar sero-flocculent evacuations, the intolerable anguish, oppression, and feeling of intense burning heat in the epigastrium, the ardent thirst, and paroxysms of excruciating griping pains in the bowels, nausea and retchings, mark an affection of the mucous membrane of the alimentary canal. If no morbid change of structure, or striking alteration of vascularity, is present in every case to explain the severity of the symptoms, this is not contrary to our experience; in other diseases, changes of structure are sequelæ, not causes, of disordered vital actions, of which mere vascularity forms the least important part. Thus, in hooping-cough, and various other local diseased conditions, the natural sensibility and sympathies are highly exalted, without any perceptible alteration in the fulness of the vessels of the part, and little augmentation of sensibility is often found in conjunction with a highly-distended state of the capillaries.

The failing pulse, the coldness of the surface, the livor and shrinking of the external parts, and some portion of the oppression of respiration, belong to disordered functions of the circulating system, in which there is a remarkable failure of the heart's action, and equally remarkable *spasm of the capillaries*, combining to arrest the circulation, and the changes which take place in its natural course; the animal heat ceases to be generated in a great measure, so as to be diffused by the languid circulation; the very breath becomes cold; and although an internal burning is complained of in the bowels, the same is also frequently the case with the surface, the patient tossing off the bed-clothes from a sense of intolerable heat, when his limbs are cold as marble. The blood becomes purple and exceeding thick; there is a rapid transudation from the vessels of the skin, and even from those of the brain. There is likewise an affection of the muscular system, spasms of various degrees of intensity, from the slightest cramp to an agony little short of tetanus. By this the muscles of the abdomen and the diaphragm are frequently affected, augmenting the difficulty and oppression of respiration.

With the exception of ringing of the ears, head-ache, and some few other symptoms too unimportant to require notice, the only affections of the nervous system are, giddiness and deep prostration, arising from exhaustion, pain, the disorder of the heart's action, and changes which take place in the blood.

Of these disordered functions of different classes of organs, the primary and most essential is that of the mucous membrane of the alimentary canal: the spasms may be wanting or severe; the asphyxia may be excessive or absent, little more affection of the circulation than that from an ordinary purgative taking place; but this is always present in the almost infinite variety of forms and grades assumed by this epidemic. Viewing it always preceding these other symptoms for a longer or shorter interval, and knowing the heart's action to be so powerfully influenced, both in the healthy and diseased state, through impressions received by the mucous membrane of the stomach and bowels, of which sinking during abstinence, and rising and quickening after food, palpitation from irritation of the stomach, and becoming exceedingly weakened and retarded by the paroxysms of pain in colic, are instances: noting the resemblance of the phenomena of this disease to the operation of an emetic, and particularly the observation of the total failure of the pulse, *following directly upon the accession of a paroxysm of severe pain:* we can have little hesitation in attributing the failure of the heart's action to this cause; if the effect is great, the agent is violent and fully adequate to produce it. Indeed, a pain equally severe in any part of the body, independent of the powerful sympathies existing between the stomach and the centre of the circulation, is capable of producing syncope in most people, and some are thus affected by very slight causes. The oxygenation of the blood becomes imperfect, and it acquires a very singular inspissation. Some have thought that the cause of the latter phenomenon was to be found in the drainage effected by these profuse secretions. It has been asserted to be deficient in its saline constituents, which abounded in the evacuations; this is too delicate a matter to be decided from the small number of experiments yet performed; and, with regard to the drainage of watery particles, we are bound to confess that it is, in a great measure, contradicted by other observations. Evacuations the most profuse, such as the bucket-full, *suddenly* discharged in common cholera, are often attended with very little of this effect; and it has no uniform relation to the quantity secreted, whether retained in the bowels or discharged, in cases of the present epidemic. Particular observations made *here*, with a view of throwing light on this question, have not been published; but, from the descriptions of cases we have given before, and from all which have been published by others, it appears that Mr. Scott's remarks apply to the epidemic in this country, as well as to that of Madras. After observing that the quantity of clear fluid discharged is sometimes very great, and, if it were to be uniform, it would readily explain the weakness, thirst, and other symptoms; he adds, " nevertheless it is unquestionable, that the most fatal and rapid cases are by no means those which are distinguished by excessive discharges. That these discharges must diminish the serum of the blood is certain; but, from observations made on the blood in highly-excited states of the circulation, in which it is found that the fibrine becomes actually more fluid than the serum, diluting the whole blood, whence it finds a more ready passage through the capillaries; also the fibrinous portion becoming thus more readily transfused. From this fact, taken together with the above, it is highly probable, if not absolutely certain, that this inspissation of the blood arises *chiefly* from some change in the state of its fibrinous constituent, induced by the languid circulation, and probably influenced by the shock on the nervous system. After death from blows on the stomach, the blood remains fluid; likewise after fatal mental emotions, electricity, and many other agents. *The blood no longer possesses the power of acting on the oxygen of the inspired air to the usual extent.* From the obscurity which at present involves the physiology of this function, we can at present offer no further explanation, than by alluding to the close connexion which exists between the different parts of the body. A severe injury cannot be done to any one organ, and particularly to those of the nervous system, nutritive, or circulating and respiratory systems, without greatly disturbing, if not altogether, for a time, overturning the functions of the rest. The oxygenization or decarbonization of the blood is certainly, of all, the most essential to life, and the most independent of all of them, but assuredly not beyond their influence; and the fact remains the same, whether the agent connecting them in this close union be the nervous power, or some other thing, at present concealed among the mysterious phenomena of life."

*The Constriction of the Capillaries.* The function of these vessels remains, for some time after the death of all the rest. When the heart is enfeebled, or ceases to act, they carry forwards their contents and contract, inducing the physiognomy so well described by Hippocrates; and there does not appear to be any reason to believe this organic contractility to be augmented, in any extraordinary degree, in cholera This apparent spasm, to which we have before alluded, appears to be referrible to long prostration of the heart's action, and the derivation of the blood to the abdominal cavity.

In alluding to the different varieties of this affection of the circulation, we noticed the fact, that some persons are more readily influenced by pain and other agents than others. We have known children faint at the sight of blood, and many persons from the slightest pain, while the power of endurance in others is almost incredible. This fact may assist in explaining some of the cases of sudden prostration which occasionally take place in persons apparently very robust without a proportionate increased severity of the abdominal affection; but the majority of persons *so seized* are of a present health and constitution debilitated by various causes; by previous sickness, long habits of intemperance, a previous day's debauch, excessive fatigue, sudden grief and anxiety, deficient nutrition and protection from the seasons—all agents rendering the heart's action liable to be prostrated by slight causes. And we also find that atmospheric influences, in some unknown way, so operate on the body that very slight excitement or local irritation will powerfully disorder the whole functions of circulation, and the healthy changes which take place in the blood. There is reason to believe that this influence has greatly contributed, also, to modify the forms of this disease, particularly in relation to the intensity of the effect produced on the circulation by the affection of the mucous membranes, both in the stage of collapse and consecutive fever. Thus, we find this fever prevailing more in some particular localities, and taking on the type of the prevailing one of the season; being in Bengal of a bilious remittent form, and typhoid in parts of Russia and England.

There appears reason to refer the spasms of the voluntary and respiratory muscles also to the disorder of the mucous membrane. They seem to be most violent the nearer this approaches an inflammatory character. In India they were observed most particularly amongst the Europeans, and it appears that bleeding was of most service in those cases where they formed the prominent symptom, as in Dr. Burrel's practice; and from the same observations by others we have before observed, that some enquirers found in dissections of patients where they had been most violent, a bright arterial injection of the mucous membrane, but it has not been ascertained to be an invariable connexion. They exist in common cholera in equal intensity, and there does appear, from the observations of Baron Larrey on Tetanus, and the prevalence of this disease in Barbadoes and other torrid climates, that peculiar influences of the atmosphere do induce a state of the system in which the slightest local irritation, whether of a wound or disordered contents of the alimentary canal, are capable of exciting the severe and mysterious disease, tetanus.

The contraction of the bladder is in the healthy state induced by chills on the skin; it also is connected with the bowels, being supplied by nerves from the same origin. In females pregnant at the time of seizure, abortion is observed invariably to take place. Abortion likewise is frequently produced by violent purgatives, and may be referred to connexion through sympathies with the stomach and bowels.

" We come, lastly, to the consideration of the nature and production of this peculiar affection of the mucous membrane of the alimentary canal. The whole of its functions are greatly disordered—secretory, sensible, and motive. For the seat of this disorder some look to the mucous membrane itself; others again, recollecting that in various affections the symptoms are referred by the patient to the sentient extremity of the nerve, although the diseased action occupies its trunk or origin, as in painful affections of the nerves of the face, and some cases of paralysis, where pain is experienced in the extremities—and finding the mucous membrane often pale, and to all appearance natural, it is no wonder that they should be led to seek its seat in an affection of the nerves by which these viscera are supplied. Dr. Loder of Moscow first broached this theory, and Dr. Delpech has lately supported it by dissections. In some few cases he has found an unusual hardening with or without infiltration and injection of the semilunar ganglia, the solar or renal plexuses, inferior portion of the pneumo-gastric nerve, and sometimes even the pneumo-cardiac plexus. He has displayed great ingenuity in deducing a ratio signorum from these appearances; but until the occurrence is found to be invariable, and the few cases at present examined by no means warrant this conclusion, we must be allowed to believe that the sentient extremity, not the origin or course of the nerve, is involved in this peculiar diseased action. Whether evacuated or not, the only constant and invariable occurrence in all cases of the disease, is the production of the secretion; and we have before shewn the error of that theory which supposes that injection of the capillaries with red blood must form a necessary part of all diseased actions where sensibility is highly exalted, because such is the case to a certain extent in inflammation. Besides, our knowledge of the pathology of the sympathetic and its ganglia is not sufficiently advanced to deduce any clear ratio signorum from such appearances."

By those who, like ourselves, consider the seat of this disease to be an affection of the mucous membrane, different theories have been invented to explain its origin; these we may divide into two classes. Some of these persons observing in the action of poisons upon this mucous membrane, previously in a healthy state, effects greatly like the symptoms of this disease, have put forth a speculation that it owes its origin to a poison generated by chemical changes in the matters contained in the stomach and bowels, (as an illustration of his ideas on this subject, one writer refers us to the production of a poison in German sausages—Dr. Ruck.) In this theory the affection of the mucous membrane is considered as secondary—the result of the action of this poison upon it. Other persons, taking for their guide the excessive disturbance of the functions, sensibilities, and sympathies, of different parts which take place in their local diseased conditions, as in various inflammatory, catarrhal, and other affections of different organs, have considered this diseased state of the mucous membrane as the primary affection, the cause of which some have believed to be a specific poison received from without, existing in the air, as malaria, or generated by the sick, and transmitted from one person to another, as the virus of small-pox and other diseases. These animal poisons have specific effects on different organs, as the contagious exanthemata, itch, and porrigo on the skin, that of hooping-cough on the membrane lining the air-passages, and the supposed poison of cholera on the alimentary canal. Others again observing diseased states of different parts of the body induced by the variations in the humidity, temperature, and electricity of the atmosphere, as pneumonia, pleurisy, catarrh, diarrhœa, fevers, and a great number of other maladies, have believed that this peculiar affection of the alimentary canal has been produced by the same influences, of whom the greatest number actually believe it to be a catarrh—that of the pulmonary mucous membrane is indeed accompanied by some constitutional affection marked by *excessive* sensibility, and consequently great disturbance of the parts sympathetically connected with this sensibility, namely, the muscles of respiration which are frequently thrown into convulsive action, and there is also an abundant pale secretion.

Of these different theories we may observe, that not a shadow of proof has been advanced for the production of a poison by chemical changes in the contents of the bowels. That of contagion we have found it most convenient to examine before. The existence of miasmata is unsupported by facts. That there is a peculiar affection of the mucous membrane is certain, and for reasons already given we believe it to be the proximate cause of the disease; but whether it is in its nature the same as the pulmonary catarrh, or some other affection sui generis, we must leave to be demonstrated by future observations. The occasional rapid attacks, and still more the rapid recoveries, are contrary to the course of *catarrhs*. The manner in which the sick describe their feelings is vague, and it is very difficult from them to ascertain the exact order in which the symptoms succeed each other. The following minute description of an attack of cholera by a medical friend who has suffered several times from the disease is of great value in these relations.

"For some days previous to each attack I experienced a slight degree of languor and dejection of spirits, particularly after dinner, which increased towards evening. I continued to eat as usual, but food lay rather heavy on the stomach. On the evening preceding the attack these feelings were somewhat augmented, accompanied by an unusual though slight sensation of irritation and load *in the bowels*, which increased towards night, and on the last occasion much offensive wind was discharged. After going to sleep as usual, in the middle of the night I was awoke by an indescribable augmentation of the uneasiness apparently throughout the whole track of the alimentary canal, with much load and oppression. I felt that if I could at once get rid of its irritating contents, I should experience great relief; (the same feeling that occurs to a less extent when nausea and vomiting is produced by food which offends the stomach.) Nausea and pain of the bowels rapidly came on as the action to expel their contents commenced. The pulse became low, the skin cold and constricted; and an indescribable prostration of strength came on with the first accession of griping pain and nausea, and these effects were increased on each fresh occasion—the peculiar feeling of great uneasiness augmenting each minute. In the course of half an hour the stomach had been unloaded of undigested contents by vomiting, and the bowels of very offensive fæculent stools—at first consistent, then more and more fluid as secretion took place, until they became quite watery. The feeling of load and oppression was now removed, but the accessions of spasms in the bowels and sickness continued to recur, although at longer intervals, when some fluid was discharged of a pale colour, in which subsided small portions of whitish flocculent appearance, leaving the supernatant fluid semi-transparent. There was very great thirst and feeling of heat in the bowels; but the stomach would not retain anything. When the sensation of a load in the bowels had been removed, and the paroxysms of pain became slighter, and recurring at longer intervals, the severe coldness of the surface diminished, and at length was followed by some degree of heat and moisture although readily chilled. These symptoms, with thirst and heat in the bowels, remained after the griping and purging had subsided—the only thing which now seemed to be borne by the stomach was tea without sugar or milk—its astringency was grateful, while the idea of any alimentary substance excited nausea. Slight purging of the same fluid continued through the day, but gradually declined. The weakness abated, and towards evening the stomach became capable of receiving a very small quantity of light bland food. The first subsequent fæculent evacuations of the next day were deeply tinged with bright orange bile. The cramps which occurred in both attacks were slight."

In this case the succession of phenomena was first weakness of digestion, induced by atmospheric influences, increasing until this function ceased to be performed—the mucous membrane became exceedingly irritable, taking on suddenly a violent spasmodic action to expel its disordered contents; after the expulsion of which, the commotion excited remained for a short time, as it does after a severe purgative, and then gradually subsided. The whole of the premonitory symptoms were however so slight, that they would probably have escaped the notice of most common observers; and the general appearances which constitute the chief part of the descriptions of others, could not be noticed by the patient. Here was a peculiar disorder of the mucous membrane, aggravated by the irritation of its contents, and it does appear to us, that the phenomena and causes of the present cholera, although infinitely various in degree, are of the same nature.

### Treatment.

In each country where this disease has appeared, we find a very different and opposite treatment recommended, as one proved by experience to be the best. In India, bleeding, calomel, and opium, were the favourable remedies. In Russia, a practice as inert as a few grains of the subnitrate of bismuth, in frequently repeated doses. In England, the mustard emetic. Again, in different countries, remedies contradictory as bleeding and transfusion, are proposed and made use of in full confidence. Heat applied to the body in every form, as well as the cold affusion. Drinks altogether denied, or ordered in unlimited quantity, both hot and cold. They have attempted to restrain purging and vomiting by the most powerful narcotics, enormous doses of opium; and they have also encouraged them by various emetics and purgatives. Some try to allay the irritability of the mucous membrane; others goad it with the most powerful stimulants, ardent spirits and ammonia—others place their reliance chiefly on the mild alkalies, soda and magnesia, to neutralize some imaginary agent; and, again, an indication has been found for acids: whilst many have trusted a trifling carminative, as essence of mint, or cajeput oil, to combat these alarming symptoms;—illustrating the remark of Sir William Crichton: "it is a most melancholy confession, but one not the less true, that after cholera has spread its devastations from Ceylon to Archangel, from Orenburg to Berlin, we are almost as far from a rational methodus medendi as we were when it first appeared on the banks of the Ganges."

Of all this false experience the cause is the same as that which, in every age, has formed the chief obstacle to the progress of practical medicine; namely, the difficulty of knowing what would have been the course of the disease, if left to the operations of nature; and what part of the effect, whether good or bad, is correctly due to the treatment employed.

In our critical examination of the treatment employed in this country, it is necessary to bear this axiom in mind, and as an antidote to overweening partiality for the remedies made use of here, let us recollect the misplaced confidence of others.

Many a valuable remedy has been first suggested by a stupid or erroneous indication, therefore, it will be well to ascertain the practical effects of different remedies before enquiring into the principles which have directed their use.

### Blood-letting.

This remedy requires the earliest notice, from the very great extent to which it has been used, as well as from the very favourable reports of its efficacy, from almost every country where the disease has appeared. It is unquestionably the most powerful agent made use of, whether with the intention of relieving congestion or removing the irritability of the stomach and bowels; but at the same time it is a remedy, unfortunately, capable of producing equally injurious consequences, if misapplied: an instance is given, in the case of Mrs. Fairly, before related; she was suddenly attacked by the disease, at 11 o'clock, a. m., Dec. 7. and had pills containing calomel, capsicum, and opium, immediately; at 12 o'clock she was bled to about 20 ozs. (Mr. Parson's report), after the bleeding, the pulse almost completely disappeared at the wrist; the symptoms of oppression, which had appeared just before the bleeding, rapidly increased, and she died in a little more than nine hours from the commencement of the attack, unrelieved by the assiduous application of brandy, cajeput oil, and a strong solution of the subcarb. ammoniæ; copious hot injections, the first of which contained two drachms of laudanum; frictions with hot flannels, and a sinapism to the stomach. We might bring forwards a number of other unfavourable instances, but this will be sufficient. The important question is, what are the circumstances under which it is beneficial: we regret that we are at present unable to give to this any satisfactory answer: the cases in which it was so successfully employed by Dr. Burrell, were remarkable for the severity of the spasms; and in the dissections were observed, great congestion of the veins of the abdominal viscera, and a highly injected state of the mucous membrane of the stomach and bowels.

In the very valuable report of Dr. Gibson, on the treatment of cholera in the North of England, it is observed, that where, in the second stage, the spasms are severe and the pulse strong, bleeding should be practised as a general rule; but that several cases have run a rapid and fatal course, which were thus favourable to its use. In the early stage he recommends it also in young and robust subjects, especially where there is pain in the epigastrium. Other practitioners unite in expecting benefit from it in the very commencement of the disease, and here we must point out a source of error in estimating its value, and a shameful abuse of the remedy.

Since diarrhœa forms the most frequent premonitory symptom of cholera, and no one can tell when the vomiting and collapse will supervene; since the number of these cases which do not terminate in cholera is greater than those which do, and of the latter but a small proportion take on the form of severe sinking of the heart's action; it is evident that the practitioner will erroneously infer, that bleeding has arrested the disease in those cases not terminating in cholera, and in those that do, he will have no suspicion that bleeding may have determined the event. Moreover, it is in the severe cases where a remedy is chiefly wanted, the slight ones will get well of themselves; and there are, indeed, unhappily, very few cases of cholera in which we can truly say, that any remedy has snatched the patient from the jaws of death. The following quotation from Riverius, who flourished at Montpelier, before the time of Sydenham, is a literary curiosity, even if it did not so *remarkably agree* with the results of modern experience. After remarking on the danger of relapses, which occurring when the patient seems out of danger, not only deceive the uninitiated, but even sometimes the physician himself; he observes, this danger is to be averted, not only by restoring the strength by remedies before proposed, "but especially by blood-letting, which most powerfully *derives* and calms the hot and raging blood, and this is to be repeated twice or three times, if the strength is increased rather than lessened by the first. Yet some practitioners dare to bleed on the first attack, even when the strength is most prostrated, which they say is oppressed, not really weakened; but at this time blood-letting cannot be performed without danger, for the sick are sometimes suddenly cut off shortly after the use of this evacuation, not without bringing discredit on the remedy."—*Opera Omnia*, p. 287.

### MUSTARD EMETICS.

The use of this remedy was first suggested by Dr. Gibson, and has been frequently employed in the North of England and Scotland, to such an extent, that it has been customary to give it in any bowel-complaint of the season, for fear that the symptoms of cholera should come on; although so great a favourite and so shamefully abused, we cannot find that it has come into general notice from any superior effects to the many other remedies which have obtained an ephemeral notoriety. In the first case, published by Dr. Lindsay, in the Cholera Gazette, the man had been ill 12 hours before he was brought to the hospital, *but* the skin and tongue, though cold, were not remarkably so, neither had the surface as yet assumed a livid hue; the patient also could still be roused, when spoken to, and pressure upon the epigastrium shewed he was still sensible to pain;—expressions which *imply*, without asserting, a more violent collapse, than a direct statement would admit of. Two drachms of mustard mixed with about eight ounces of warm water were got down, and for about 10 minutes a copious vomiting took place, after which the pulse was to be felt at the wrists, and other parts of the body, even soft not feeble; but, in about 15 minutes after, the patient was attacked with cramps, so violently, that he uttered piercing cries, and thrust his feet towards a large stone near which he lay in the hope of obtaining some relief from the pain he was suffering. When the violence of the paroxysms, for it was truly such, had in some measure subsided, the patient was replaced in bed, and though still suffering severely from acute pain, and evincing by his cries and his actions *a strong degree of vitality*, it was observed with surprise, that the *heart's action had* apparently ceased, and that no pulse was to be detected at the wrist, or any other part of the body. As the cramps ceased the patient became again quiet, and gradually assumed the ghastly appearance he had presented on admission. It was determined, therefore, to repeat the mustard emetic, and this measure, on being carried into effect, was again followed by similar results, namely, copious vomiting, and after that, restoration of the pulse, and of the natural colour of the lips. The warmth of the surface was maintained: at night, 40 grs. tinct. opii, and 10 grains of calomel were given: on the following day the pulse was 80, soft, distinct, and countenance natural; but he died on the fourth day of consecutive fever. This man did not die in the first stage, it is true, but the case was never one of severe collapse. In others, it has failed to produce even vomiting, adding greatly to the agonies of the patient.

Copious injection of warm water into the rectum is another of the remedies particularly recommended in this country, and much benefit has been produced by it. The injection of oxygen gas was also suggested by Dr. Hunter of Edinburgh; but, besides a temporary excitement, no permanent beneficial effect has been obtained. The same observation applies to the inhalation of oxygen gas; in all cases where it has been tried, it has produced a well-marked temporary excitement, but no permanent benefit, nor was any other to be expected from it. The symptoms do not arise from any deficiency of oxygen in the atmosphere, or *deficient respiration,* but failure of the usual influence of the blood on the air.

Tobacco enemata have been used; the statements of their effects are contradictory. Mr. Caton observes that, in a stout man, in whom the symptoms of collapse and spasms were severe, and unaltered by the use of external heat, the mustard emetic and other stimulants, an enema of three drachms of tobacco, in half a pint of water, was followed by flushing of the countenance, moisture and warmth of the skin, and return of pulse proceeding to recovery. Several other severe cases have done well under its use, but in others it has failed, in some being followed by sudden death Of various stimulating remedies, such as the subcarb. ammoniæ, camphor, brandy, Cayenne pepper, there is little worth notice to add; they have been given in many shapes and variously combined, with no certain benefit, for in those cases where a remedy is wanted, they fail.

So much for the treatment of symptoms. When we reflect that this practice is directed to the effect, not to the cause, and that the mortality of the disease, on the whole, varies from about one-third to about two-thirds of the number taken ill, we shall see reason to infer, that it has been little influenced by any treatment recommended. If an empiric remedy had been discovered, there is no person by whom it would be more valued than by ourselves; but, until this is the case, it is our duty to direct our efforts to deduce a correct ratio medendi.

In our examination of the disease we have supported the opinion, that its proximate cause is a peculiar affection of the mucous membrane of the bowels, and that the rest of the symptoms are produced by sympathies existing between this membrane and the rest of the system. It is the presence of food in the stomach and chyme in the bowels, which, in the healthy state, excite their secretions and motions; and, in all their disordered states, the first and most powerful means which we can employ to quiet their diseased actions, is to obtain the evacuation of their contents. We, therefore divide this disease into two periods in relation to the treatment; the first previous to the expulsion of the whole of the usual contents of the alimentary canal, the second after this effect has taken place. Whilst a particle of foreign matter remains, the most important indication is to obtain their expulsion in the speediest and easiest manner possible. If symptoms of a loaded and oppressed stomach exist, by emetics; if otherwise, by purgatives. In the former class, the sulphate of zinc, solution of salt, mustard, tartar-emetic, subsulphate of mercury, and ipecacuanha, have been used, although often from an indication somewhat different; and, amongst the purgatives, there is scarcely any one which has not been employed. It is a question, whether the occasional benefit derived from calomel is not owing to its operating in this manner, although scarcely ever directed with this view, and generally in the second period, when the indication no longer remains. Castor oil has received commendations as great, from those who have employed it, as any other remedy has from its warmest admirers.—*See Edinburgh Medical Journal for* 1826.

From the history of cases in which various emetics have been used, there does not appear to be any proof of superior efficacy in any particular one, if we except Professor Reiche's use of tartar-emetic; and yet the extraordinary results of his practice require confirmation from other hands. The emetic best adapted to the milder cases is certainly ipecacuan, at once safe, speedy, and certain in its operation; and, in the more severe cases, none have yet been found better than this, or the tartrate of antimony. The expulsive powers of the stomach, in some cases, become so far prostrated, that they cannot be roused by any agent: here it is our duty to refrain from increasing the sufferings of the patient.

It is in the premonitory stage of diarrhœa, that opportunity most frequently occurs for the use of purgatives, and none has been yet found superior to rhubarb, of which a single dose of half a drachm, in powder, with five grains of ginger, and one ounce each of brandy and water has the best effect, when the bowels yet retain a considerable quantity of fæculent matter; but, if the major part of this has been evacuated, the strained infusion of the same quantity in two ounces of water, with a scruple of carbonate of soda and magnesia, is to be preferred. On the first accession of the cholera, when it makes its invasion suddenly, those only who, like ourselves, have taken the former remedy, or a full dose of the compound tincture of senna, can have any conception of the indescribable relief they afford. In the milder cases, the contents of the alimentary canal having been expelled, there is no further occasion for any other remedy, for a time at least. All are productive of mischief. The extreme irritability continues a short time; paroxysms of pain recurrring, as the accumulation of even these bland, secreted fluids proves a source of irritation; but they recur at more distant intervals, until they cease altogether. The thirst requires to be quenched with fluids containing no alimentary matter; and all food should be avoided, until the healthy functions of the mucous membrane return, and then admitted in the most sparing quantity only.

Along with these remedies, the copious imbibition of watery fluids is beneficial; if they are rejected by vomiting, it does no harm; if they are not, they dilute, and assist the expulsion of the contents of the alimentary canal. It is far otherwise when great irritability remains after their contents are cleared out, then the less that is given the better; and again, when vomiting has ceased, but the thirst is extreme, they may be with benefit indulged in; the patient's own instinctive feelings being generally our best guide as to what liquid may be given with most benefit.

The benefit experienced from the use of these means will be greater the earlier they are adopted; and in those cases where they fail, no remedy at present discovered will succeed; the nervous system has received a shock, and the functions of life are arrested; we may prevent, but, like that of concussion of the brain, it will depend on other circumstances than our efforts if a favourable change succeeds.

From the known effect in colic, from the frequent contraction of the colon which takes place in this disease, and from the benefit which has resulted from its use, injections of warm water are valuable at any period of the complaint, but they must be used in great quantity to be productive of benefit.* Blood-letting, if we may judge from general experience, affords more relief in those cases where the symptoms of irritation predominate over those of prostration.

The important question is, what are the circumstances under which it is beneficial? We regret that we are unable to give to this any satisfactory answer. We earnestly recommend the subject to the attention of the many diligent enquirers now investigating the best treatment of this disease. It is customary to cite the practice of Dr. Burrel in India as a proof of the efficacy of bleeding. He states that out of 88 cases that were bled only two proved fatal.

In the last period, when the bowels contain nothing but their own secretions, we have especially to beware the nimia diligentia medici: great irritability in the mucous membrane still remains, and the effect on the nervous and circulating systems has taken place. It is easy to do irreparable injury, but difficult to produce any certain benefit.

### CAUSES.

Besides contagion, there is none of the other known causes of epidemics which can have prevailed so offensively as distemperature of the atmosphere. At the present time we have not space to enter fully into the consideration of this question, which is indeed one of inferior importance to those of the nature and treatment of the malady. Neither have a sufficient number of facts been at present collected, to enable us to decide either for or against it. All that we can do now, is to expose many errors which have been committed in reasoning on this question, and to point out the proper course to be followed in the inquiry.

---

* In a case of colic which had for three days resisted all other means, purgatives aggravating the complaint and bleeding affording no relief, the disease was at once subdued by injections of warm gruel. Six pints were slowly injected, with the sensation of reaching the place of the stricture, and giving some relief; it returned in a short time unchanged. Immediately nine pints were slowly injected, this quantity being all that could be borne—from this very great relief followed, and it was allowed to return, bringing with it a small quantity of fæculent matter. A similar quantity was in the same manner repeated, and the pain entirely ceased—a full dose of opium was given—it did not subsequently return. The first evacuations were scalding and dark green.

The suggestion that the cause of this disease may be found in distemperate conditions of the air, was first derived from the belief that other epidemics are produced in this manner. Observation has shewn that pneumonia, pleurisy, and catarrh, are certainly often produced by the exposure of the body to the vicissitudes of the air; and in different seasons there prevail different diseases. With the rising temperature of Spring appear cutaneous complaints; with the declining temperature of the latter end of Summer and Autumn, affections of the alimentary canal come on; and when the cold becomes more intense, the respiratory organs suffer the most; catarrhs, pneumonias, and pleurisies are more frequent; and it is also remarked, that different years are dissimilar both in atmospheric conditions and in their prevailing diseases. What is the wonder, then, that the cause of the latter should be sought in the sensible qualities, temperature, moisture, and electrical condition of the former, especially since there is no other known agent to account for this effect? But here commence the difficulties of the enquiry. Our venerated Sydenham remarks that, "although he had observed, with as much diligence as possibly he could, the various dispositions of divers years, as to the manifest qualities of the air, that from thence he might learn the causes of this great variety of epidemic diseases, yet he received no benefit thereby; for he perceived that years which do agree as to the manifest temperature of the air are infected with various diseases, and so on the contrary." The thermometer was not yet come to sufficient perfection to afford him its unerring standard of comparison of different temperatures; but another observer, who had enjoyed its advantages, does not differ in his conclusions. Dr. Bateman observes, with regard to epidemic febrile diseases, "that no plausible cause has yet been assigned for their absence or prevalence; meteorological observations have thrown no light on this subject; and chemistry has not detected any change in the state of the air by which they might be explained:" and the observations of all other writers on this subject are to the same effect. Epidemics of similar characters appear when the present state of the air differs greatly in all respects, and the same present condition is not followed always by the same effects. In what are we to seek an explanation for these anomalies? Turning to the prevalence of particular diseases in different seasons, we find that, although catarrh, the most common of all maladies, is more frequent in Winter than in Summer, that single cases are to be found the whole year round, and that sporadic cases of almost every disease, apparently produced by atmospheric causes, are to be met with under every variety of condition of the air, and, on the contrary, in the severest of all non-contagious epidemic visitations, a certain number of the community have escaped. From these we learn that this complicated frame is influenced by a variety of circumstances, which enable the constitution to bear up against great vicissitudes of the atmosphere, or render it susceptible of the slightest changes. Amongst these we may enumerate, original peculiarities of constitution, the effect of preceding conditions of the air on the general health, and of every other agent capable of debilitating the body, which exist combined in such an infinite variety of proportions, that it is no wonder we have hitherto failed in establishing any general laws.

The degree of the effect of any fixed atmospheric condition differs in different individuals according to constitutional peculiarities and the state of the general health; and we maintain that the argument of Sir Gilbert Blane, "if any disorder affecting a whole community arises from some noxious principle in the soil or air, it must, in the nature of things, attack simultaneously all who are exposed to it," is founded in the utter ignorance of the manner in which operate the causes of all non-contagious epidemics; and that all the remaining arguments against the agency of atmospheric influences, which are founded on the occurrence of this disease in so many different climates, and under such great extremes of temperature, are good for nothing. Do not the common cholera, diarrhœa, and dysentery occur at Archangel as well as Calcutta, at Moscow as well as Orenburg or London? and has not there prevailed, both for some time preceding as well as during the course of the cholera in England, a medical constitution of the latter end of the Summer, the Autumn, and Winter, producing bowel-complaints and fever as the prevailing diseases of the time? Were not these bowel-complaints more general and severe at those particular places where the cholera appeared? and has not the same circumstance been observed in every place where we have reports of the state of the general health during the prevalence of cholera at Riga, Moscow, Orenburg, Petersburg, Berlin, and in India? Has not the state of the weather in this country been the same as that under which such complaints are always here observed to prevail?

and have we ever been informed that the state of the atmosphere in other places differed in its general conditions from those under which bowel-complaints commonly prevailed in these places? Why should one person be affected with diarrhœa, another with mild, and a third with severe cholera, unless it be the same agent?

A. ———.

*(To be continued.)*

---

### VI.

### EXTRACT OF A LETTER FROM DR. W. H. YATES.

*Cambridge*, 23*rd March*, 1832.

THE Cholera has broken out at Ely. Dr. Haviland was immediately written to about it, and being prevented going over himself, which he fully intended doing, he requested me to go. They are cases of decided malignant cholera. I am sorry I have not time to give you particulars; but I may state the heads. From Saturday 17th to Tuesday 20th, 10 cases—5 deaths. The general duration of the disease twelve hours—one, sixteen hours—and one, only ten and a half. This last was an infant, fifteen months old. There were two others—one, four, the other five years old;—neither of the three received any medical assistance; and took nothing but tea. Then there were two old men, seventy years of age—one a thresher—the other worked on the high road. All were badly off, ill fed, ill clothed, poorly lodged, general diet potatoes. The two old men were given to intemperance—one had been a soldier. The other cases all females, except one, a youth of seventeen, and a child, four years. Neighbourhood low, swampy alluvial soil, near the river—all the cases occurring in the same district, and confined to two streets in which nuisances abound—such as stagnant ditches, common-sewers and drains.* There is a deep dyke, into which the people empty various kinds of accumulated filth, and an open privy. Wherever there was a corpse, we found it had been laid out, and the people sitting round the fire in close debate; and always plenty of visitors. Great unwillingness to bury the dead earlier than is usual—little fear—great resignation. Those who had visited their sick friends, remained well up to the moment of my departure. Those who had been taken ill and died, could not be discovered to have had the slightest communication with the sick. The medical men are unanimous in their opinion that, at Ely, the disease has broken out independent of any intercourse with towns, &c., where the disease exists; and that it is not contagious—at least such is their impression at present. There are many poor at Ely on account of the numerous charities—no manufactories.

General symptoms—dizziness, vomiting and purging of a milky watery fluid—no fæculent matter, no bile, violent cramps of legs, arms, and body—blueness—no pulse—cold clammy skin—white pasty tongue, and cold—eyes sunk—no urine—but great inclination to pass water. In fact, there can be no doubt of the identity of this disease, and that in London, but various in degree, and the dissections prove it.

A great sensation has been produced here,—my return was anxiously looked for;—I was summoned before the Board to-day (the Vice-Chancellor in the chair.) I read them a very long and detailed report of the cases, and many particulars which I cannot here repeat; it is expected the disease will find its way to Cambridge. Since I was there, I understand there is another case (dated Thursday morning.)

*Dr. J. Johnson.* W. H. YATES, M.D.

---

* Mr. Dendy, an accurate and philosophical observer, stated in the London Medical Society, on the 26th of March, that he was able to trace almost every case of the epidemic in Southwark and Lambeth to malarious localities, and especially to ditches. We are confident he is right.—ED.

### VII.

### CONCLUDING OBSERVATIONS ON THE EPIDEMIC.

The more we have seen and learnt of the reigning malady, the more convinced we are that the observations made in the last page (304) of our last number, are correct:—namely, that the epidemic is a choleric fever, the first stage of which is diarrhœal (or gastro-enteric); the second, asphyxial (or congestive); and the third, typhoid—or inflammatory. The first or diarrhœal stage has prevailed for many months, and throughout a large range of society. In its milder grades, this diarrhœa is often no more than some derangement of function in the stomach and bowels—and is—the cholera of the rich. In the stage of regular diarrhœa, it is the prevailing malady of the middling classes who are predisposed to it by diet, habits, or idiosyncrasy. Among the poor and intemperate, it too often runs on to the asphyxial stage, from which, if they emerge, they encounter another trial for life, in the typhoid or symptomatic fever which almost always follows.

But while we acknowledge the operation of some general epidemic cause, totally unknown, as to its nature, we cannot but observe the facility with which the disease springs up in certain malsain localities, and the tenacity with which it clings to them afterwards:—thus presenting far more of the endemic than of the epidemic character. The right bank of the Thames, which was, not many years ago, a marsh, and is now the chief seat of disease, is a sufficient example. The same partiality for the banks of rivers and unventilated alleys has obtained generally in England—the same preference for the lower orders of society. Does it here mow down bodies of athletic soldiers as in India? Does it attack more of the nobility than of the mobility (proportionately) here, as it did in some parts of the Continent?—No.

In respect to the treatment, we would offer a few hints. The diarrhœal stage is easily managed, where common comforts and proper medicines can be procured. Where these are wanting, the second stage will too often be superinduced, and prove intractable. In this second, or asphyxial stage, we would again admonish our brethren against the rash application of the Indian practice, employed, be it remembered, among robust European soldiers, who were not reduced by diarrhœa or enfeebled by bad food and poverty previously. We have reason to know that most of the experienced practitioners in Southwark and Lambeth, have now relinquished the attempt to restore reaction by bleeding in the cold stage. We certainly should prefer moderate excitation or an emetic, leaving Nature time to set up the balance of the circulation, as in cases of bad intermittents, rather than drain away the few pounds of blood already left in the body. We would advise as much cool drink as the stomach will bear, with a large dose of calomel to allay gastric irritability, and to prepare the way for restoration of secretion.

When reaction is established, we have a typhoid fever to encounter—and not seldom topical inflammation, especially about the head. Here the utmost care is necessary, not to stimulate too much by food or physic, under the impression of a name—typhus, or debility. Every organ and its function should be carefully watched, in order that the seat of local disease may be detected. The guarding against topical inflammation, and attention to the intestinal discharges are almost all that is necessary in the consecutive fever.

The question of contagion will, we apprehend, resolve itself into the proposition which we have long maintained—namely, that this attribute of fevers in general, is not an essential or primary character, much less a cause, of the epidemic. It is a contingency, capable of being *produced* by crowding, filth, and concentration of effluvia from human bodies—capable also of being *prevented*, or *dissipated*, if formed, by ventilation, cleanliness, space. In the diarrhœal and asphyxial stages, we cannot believe it to be contagious—in the typhoidal or febrile stage, we see no reason why it should not be as contagious as fevers of a similar type—nor do we see reason for considering it to be more contagious than they.

Every case we see, every narrative we read, impresses us, more and more, with the influence of *locality*, in the production of the disease. As far as London is concerned, the banks of the Thames are the throne and seat of cholera and malaria. Look at the place (Ely) where it has broken out in Cambridgeshire. The poor are its victims ; but predisposition and locality may reduce a peer to the condition of a pedlar or pauper, so as to take on the disease. This day (23rd of March) a lady died with all the symptoms of the prevailing epidemic. She lived in a locality peculiarly malarious, and where we have repeatedly seen intermittent diseases. She was seized with diarrhœa on Wednesday, and did not apply for medical advice till one o'clock on Friday morning, when the spasms and vomiting had come on. At five o'clock, the collapse supervened on the spasms and vomiting. At nine she expired. She was a weakly female, and the disease, we have no doubt, might have been easily arrested, if it had been taken in the diarrhœal stage. Intermittent forms of the disease are daily presenting themselves to medical observers.

*27th March*, 1832.

---

### VIII.

### ADDRESS TO THE MEDICAL PROFESSION, AND TO THE PUBLIC GENERALLY.*

THE present alarm respecting the disease which is now said to exist in London and its vicinity under the name of Cholera, is maintained by some to be unfounded or exaggerated ; while by others it is asserted, that this alarm is neither without cause, nor disproportionate to the danger. Whence such diversity of opinion may have originated, has not been ascertained ; nor has it been discovered whether there be any reasonable cause for the prevalence of opinions so opposed.

This unsettled state of the public mind has been productive of consequences the most pernicious. Our national relations have been injured ; our internal prosperity has been shaken. The medical profession have been aspersed by one party, as encouraging panic without cause ; and by another, as concealing from the public eye the real nature and the full extent of the disease.

To clear the character of the medical faculty of these aspersions by enquiring into the actual causes of this general alarm, and to reconcile these conflicting opinions, a Medical Association has been formed, to investigate the nature, modes of propagation, extent and treatment of that disease, and lay candidly before the public the results of its inquiries. As this Association belongs to no exclusive party, it advocates no exclusive views. The practitioners, of whom it is composed, espouse no theory ; they support no faction : their bond of union is the public good : their common object is the cause of truth. Their interests are promoted neither by encouraging excitement, nor by resisting just alarm. If the disease known under the name of Cholera exist in London, their object is to ascertain its extent, to elucidate its causes, to explain its management, and to restore the tranquillity of the public, by an honest statement of the truth.

An Association formed with motives so pure, and an object so important, can have no enemies but those of science and of mankind. They, therefore, look to the public for countenance, and to the profession for assistance. They more especially solicit the co-operation of the medical officers of parishes and guardians of the poor. As they intend no opposition, they anticipate no resistance. In the truest spirit of philanthropy have they been organized, and with the most rigid respect to truth and candour will all their labors be conducted. They feel

---

* Want of space prevents us from making remarks on this philanthropic Association. We hope and trust that it will soon embrace a large portion of the active, zealous, and intelligent classes of medical practitioners in this metropolis, and the country at large. From none others do we expect co-operation.—*Ed*

that the principles which they follow can lead them only to truth, and they are persuaded that the plan upon which these principles shall proceed, can scarcely fail to ensure ultimate success.

The proceedings of the Association shall at all times be open to inspection; the register for the admission of members is now prepared for signatures; and while they express the most friendly feelings towards such as may decline to join them, the Association earnestly solicit, and confidently hope for the speedy co-operation of all, who are zealously attached to the interests of medical science, and wish sincerely to promote the welfare of the human species.

LEONARD STEWART, M.D.—*Chairman.*

All Communications to be directed, post paid, to Dr. MORRIS, 17, Southampton Street, Strand, where the Register for enrolling Members lies open for Signatures.

